# Insight into the adaptation mechanisms of high hydrostatic pressure in physiology and metabolism of hadal fungi from the deepest ocean sediment

**DOI:** 10.1128/msystems.01085-23

**Published:** 2023-12-20

**Authors:** Maosheng Zhong, Yongqi Li, Ludan Deng, Jiasong Fang, Xi Yu

**Affiliations:** 1 Shanghai Engineering Research Center of Hadal Science and Technology, College of Marine Sciences, Shanghai Ocean University, Shanghai, China; Third Institute of Oceanography Ministry of Natural Resources, Xiamen, China

**Keywords:** piezotolerance, hadal fungi, development, cell structure, metabolic activity, transcriptomic analyses

## Abstract

High hydrostatic pressure (HHP) influences the life processes of organisms living at depth in the oceans. While filamentous fungi are one of the essential members of deep-sea microorganisms, few works have explored their piezotolerance to HHP. Here, we obtained three homogeneous *Aspergillus sydowii* from terrestrial, shallow, and hadal areas, respectively, to compare their pressure resistance. A set of all-around evaluation methods including determination of growth rate, metabolic activity, and microscopic staining observation was established and indicated that *A. sydowii* DM1 from the hadal area displayed significant piezotolerance. Global analysis of transcriptome data under elevated HHP revealed that *A. sydowii* DM1 proactively modulated cell membrane permeability, hyphae morphology, and septal quantities for seeking a better livelihood under mild pressure. Besides, differentially expressed genes were mainly enriched in the biosynthesis of amino acids, carbohydrate metabolism, cell process, etc., implying how the filamentous fungi respond to elevated pressure at the molecular level. We speculated that *A. sydowii* DM1 could acclimatize itself to HHP by adopting several strategies, including environmental response pathway HOG-MAPK, stress proteins, and cellular metabolisms.

**IMPORTANCE:**

Fungi play an ecological and biological function in marine environments, while the physiology of filamentous fungi under high hydrostatic pressure (HHP) is an unknown territory due to current technologies. As filamentous fungi are found in various niches, *Aspergillus* sp. from deep-sea inspire us to the physiological trait of eukaryotes under HHP, which can be considered as a prospective research model. Here, the evaluation methods we constructed would be universal for most filamentous fungi to assess their pressure resistance, and we found that *Aspergillus sydowii* DM1 from the hadal area owned better piezotolerance and the active metabolisms under HHP indicated the existence of undiscovered metabolic strategies for hadal fungi. Since pressure-related research of marine fungi has been unexpectedly neglected, our study provided an enlightening strategy for them under HHP; we believed that understanding their adaptation and ecological function in original niches will be accelerated in the perceivable future.

## INTRODUCTION

Deep-sea trenches are inhabited by abundant organisms and contain one of the largest areas on this planet (1). High hydrostatic pressure (HHP) acts as one of the key parameters in the deep sea but also becomes an obstruction for most macrobiotic species to reach greater depths. However, recent studies have highlighted the abundance of fungal diversity even in the deep sea using both culture-dependent and culture-independent approaches (2–5). In addition, evidence indicates that deep-sea fungi not only exhibit robust biological activity (6) but also play an essential role in ecological functions (7, 8). Recently, Li et al. have particularly reported the isolation of piezotolerant filamentous fungi from the hadal trenches (9). They identified 41 fungal strains that were able to thrive under HHP of 20–60 MPa and highlighted the metabolic potential of deep-sea fungi in biogeochemistry. This characteristic of deep-sea fungi has sparked our interest in further exploring the study of deep-sea fungi under HHP.

HHP impacts several crucial processes in living organisms. Prolonged exposure to HHP leads to a gradual decrease in the fluidity of the cell membrane and causes DNA damage, leading to a reduction in cell activity and eventually to cell death (10–12). *Saccharomyces cerevisiae*, a typical model fungus, has a well-understood molecular mechanism underlying its response to HHP (13, 14). The general responses include increasing the mannoproteins of cell walls, accelerating amino acid synthesis, or altering the proportion of unsaturated fatty acids (15, 16). Filamentous fungi *Aspergillus* spp. have demonstrated good secondary metabolic activity and potential to degrade the xenobiotic compounds under elevated HHP (3, 17). Deng et al. have reported that HHP regulated the production of fungal secondary metabolites (18). These studies suggest that deep-sea fungi possess distinctive metabolic pathways and adaptive mechanisms under HHP. Hence, the mechanism involved at the molecular level needs to be further investigated.

The complex ecological environment shapes diverse cellular morphologies (19). Therefore, consideration of pressure resistance mechanisms on cells should encompass various original ecological niches, rather than being limited to a single factor. Fungi display a variety of adaptations to water deprivation induced by high NaCl concentration (20). Hypersaline conditions affect the synthesis of compatible solutes and increase cell wall thickness, changes in cell membrane fluidity, ion transport, and activation of stress signaling pathways (21). Besides, halophilic fungi could withstand osmotic stress by producing substances and simultaneously adapting to a wider range of other extreme conditions (22, 23). One of these substances is known as the “organic osmolytes” mechanism, i.e., microorganisms employed organic solutes to maintain the osmotic balance inside the cell (24). These joint responses were testified by *Alcanivorax borkumensis*, in which results showed ectoine production was equivalent at 0.1 MPa in hyperosmosis-acclimated cells and at 10 MPa under isosmotic conditions (25). It sheds us a promising insight into the relationship between “osmolytes” and “piezolytes” that osmotic shocks significantly enhanced cell protection by reducing membrane damage under HHP. These results aroused our curiosity on the interaction between HHP and high salinity on filamentous fungi.

The germination of the most common *Aspergillus* species is generally divided into four stages: dormant conidia, breaking of dormancy, isotropic growth, and polarized growth (26). Due to the characteristics of intra- and extracellular structure, conidia tend to remain dormant for extended periods until they encounter favorable conditions (27, 28). Another reason for this phenomenon is conidia germination is an energy- and time-consuming and extremely heterogenic process; even though surrounding conditions are liveable, it may be delayed or dormant (29, 30). HHP severely affects cell membrane mobility, protein synthesis, and cell growth (10, 13, 31), which is supposed to be the reason why dormant conidia are usually hard to further germinate under HHP conditions. Previously, pieces of literature also have shown that spores for the deep-sea *Aspergillus* isolates are not a viable option to evaluate biomass under HHP conditions (32, 33). On the contrary, polarized growth conidia have been transcriptionally improved in growth metabolism and any other fields (34, 35) that we considered as the advantageous research subject to evaluate piezotolerance of fungi.

We believe that the lagging progress in the piezotolerance of deep-sea filamentous fungi is due to the lack of an appropriate evaluation strategy, including how to reasonably evaluate the metabolic activity of HHP-treated spores and their growth rate under various HHP. In this present work, we selected *Aspergillus sydowii* as our research subject and obtained homogeneous fungi in three vertically different origins (terrestrial, shallow, and hadal area). Then, an integrated assessment strategy of piezotolerance for two forms of filamentous fungi (dormant growth conidia and polarized growth conidia) was constructed, including metabolic activity, growth rate, and morphological characteristics. Combing with the transcriptome analysis, we might further understand the ability of piezotolerance on the molecular level in *Aspergillus* species, which gave us a promising insight into the pressure tolerance mechanism in filamentous fungi.

## MATERIALS AND METHODS

### Sample materials and isolation of fungi

Hadal fungi were isolated from sediment samples collected from hadal zone at a depth of 10,898 meters from the Mariana Trench (142.2148 °E 11.3403 °N) during the expedition of the Discovery-One research vessel (TS 21) in October 2021. The sediment samples were stored by a pressure-retaining sampling device throughout the whole process and transferred in a sterile centrifuge tube for subsequent fungal isolation experiments. The device guaranteed that there was no external microbial contamination, which ensured the authenticity of the hadal fungal isolation. Besides, we also collected the sediment samples and stored them in sterile plastic bags from the terrestrial area at Luchaogang Beach in Shanghai, China. The method of isolating and culturing fungi was followed as the method described in our previous study (18). For fungal isolation, 100 µL diluted sediments were spread plated on potato dextrose agar (PDA: 200 g/L potato, 20 g/L dextrose, 28 g/L sodium chloride, and 15 g/L agar; 100 µg/mL ampicillin), and the plates were incubated at 28°C for 3–7 days until fungal mycelium was present. The tips of hyphae were cut out and transferred to a new PDA following the assay of hyphal-tip isolation. It was repeated at least three times until a pure culture was obtained. Another strain of the same species from the shallow sea was obtained from Marine Culture Collection of China (MCCC). The growth and purification of these fungi were performed on PDA.

### DNA extraction, phylogenetic analysis, and morphological characterization

Genomic DNA was extracted from PDA-grown mycelium using the TIANcombi DNA Lyse & Det PCR Kit [Tiangen Biotech (Beijing) Co. Ltd.]. As we know that within the *Aspergillus* genus, in addition to conserved internal transcribed spacer (ITS) sequences, secondary markers could be used to ascertain the species level with higher confidence. Hence, the phylogenetic markers including the β-tubulin gene (*benA*) and the calmodulin gene (*cam*) were also picked out (36). All the primers used in this work were listed in Table S1. The protocol of polymerase chain reaction and sequencing method were consistent with previous reports (3). The amplified sequences were analyzed by GENEWIZ (Suzhou).

The identification of fungi was performed by combining morphological characteristics and conserved sequences analysis. The amplified sequences of ITS, *benA*, and *cam* were submitted to a sequence similarity search by BLASTn against the NCBI nucleotide database. The sequences with the highest similarity were selected and trimmed to construct evolutionary trees by using Clustal W in the MEGA7 software. Macroscopic features of the fungi including the color and colony on PDA and growth rate at different salt concentrations were recorded every day. The microscopic assessment was carried out by OLYMPUS microscope BX53 (Olympus Corporation, Tokyo, Japan) to observe the phenotype of conidiophores and conidia of the target fungus.

### Evaluation of metabolic activity of HHP-treated spores under atmospheric pressure

The advanced resazurin test as the previous report was used to evaluate the metabolic activity of HHP-treated spores (37). Firstly, *A. sydowii* spores were scratched from PDA by employing a minimal medium containing 1% (wt/vol) glucose, 0.5/2.0 M NaCl, and 70 mM NaNO_3_; then, spore suspension (1 × 10^5^ cells/mL) was inoculated under 0.1 MPa, 20 MPa, and 40 MPa at 28°C, respectively. After 48 hours, the spore suspension of 100 µL was replaced in a (96-well) microplate with the equivalent volume of a final concentration of 25 µg/mL resazurin. The microplate was then inoculated at 37°C, and fluorescence values were collected every 24 hours. All the treatments were replicated in triplicate. The atmospheric fluorescence value of each isolate at the 48th hour was used as the control group for significance analysis. The dead spores and pure minimal medium with equivalent resazurin were used as the negative control.

### Micromorphological analysis of polarized growth conidia under increased HHP

The fresh conidia were cultured stationarily on potato dextrose broth (PDB; PDA medium without agar) overnight to prepare the polarized growth conidia (Fig. S1). Then, polarized growth conidia were further used to evaluate micromorphological differences under the treatment with increased HHP. (1) At least 25 hyphal lengths were measured from the pressure of 0.1 MPa, 20 MPa, and 40 MPa in every 12 hours within 48 hours. (2) Mature mycelium was stained with Calcofluor white (CFW; Sigma-Aldrich, Shanghai), 4, 6-diamidino-2-phenylindole dihydrochloride (DAPI; Sigma-Aldrich, Shanghai), or propidium iodide (PI; MKBio, Shanghai) as the previous report to determine the cell wall and nucleus of hypha under different HHP (38, 39). Briefly, at least 6-day-old mycelium from the elevated HHP was stained with the fluorescent dye (1 g/L CFW; 0.5 g/mL DAPI; 1 mg/mL PI), followed by incubation in the dark for 5 min. Slides were washed with sterile water and observed using epifluorescence microscopy. Besides, dual staining with CFW-PI was also carried out to determine the viability following the previous reports (39). All staining experiments and groups were carried out at least in triplicate.

### Transcriptome assembly and transcript quantification

To perform RNA sequencing and transcriptomics analysis, *A. sydowii* DM1 was incubated at 180 rpm at 28°C on PDB until the exponential phase. Then, mycelia were transferred into different pressure conditions (0.1 MPa, 20 MPa, and 40 MPa) for 3 days, after which biomass was harvested. The mycelia were fully ground using liquid nitrogen and preserved in RNAiso Plus (Takara, Japan) at −80°C.

The RNA extraction, transcriptomics sequencing, and bioinformatics analysis were accomplished by Beijing Genomics Institute (BGI, China). According to the standard guidelines, validated RNA underwent purification, fragmentation, reverse transcription, end repairing, amplification, and circularization to get the library. Quality control was also employed. The final product was loaded on a BGISEQ-500 platform for RNA sequencing (RNA-Seq). Low-quality reads were filtered through SOAPnuke (v1.5.6) (40). Gene expression levels were calculated as FPKM (fragments per kilobase of transcript per million mapped reads) using RSEM (v1.3.1) (41). Analysis of differentially expressed genes (DEGs) was performed using DESeq2 (v1.4.5) (42). |log2 Fold-Change| ≥ 1.5 and *Q* value ≤ 0.05 were set as criteria for screening DEGs between different groups. The Kyoto Encyclopedia of Genes and Genomes (KEGG) and Gene Ontology (GO) enrichment analysis of annotated DEGs was performed by Phyper (https://en.wikipedia.org/wiki/Hypergeometric_distribution) based on the hypergeometric test.

### RNA extraction, RT-qPCR, and transcriptome validation

To verify the results obtained from RNA-Seq, quantitative reverse transcription PCR (RT-qPCR) analysis was performed as described previously (43). Total RNA was acquired from BGI, and first-strand cDNA was synthesized by the PrimeScript RT Reagent Kit (Takara, Japan) following the manufacturer’s instructions. The relative expression of genes was calculated using the 2^−∆∆Ct^ method. The RNA-Seq fold changes were plotted against the RT-qPCR fold changes to calculate correlation coefficients (*R*^2^) (44). β-Tubulin was used as a reference gene, and primers used in this work were listed in Table S1.

### Statistical analysis

All experiments and groups were done at least in triplicate, and at each sample, three biological replicates were taken. Statistical differences (*P* ≤ 0.05) among the mean of growth rate, fluorescence values, and the fold change of marker genes were determined by one-way analysis of variance (ANOVA) and Dunnett’s multiple comparisons test. The significant difference for all comparisons was set at *P* < 0.05 (n.s., *P* > 0.05, *P < 0.1, **P < 0.01, ***P < 0.001, and ****P < 0.0001). Statistical calculations were performed using GraphPad Prism 8.2.1 (GraphPad software, San Diego, CA). The standard deviation of the mean was represented by error bars in all column graphs across three independent experiments.

## RESULTS

### Identification of different origins of *A. sydowii*

**Fig 1:**
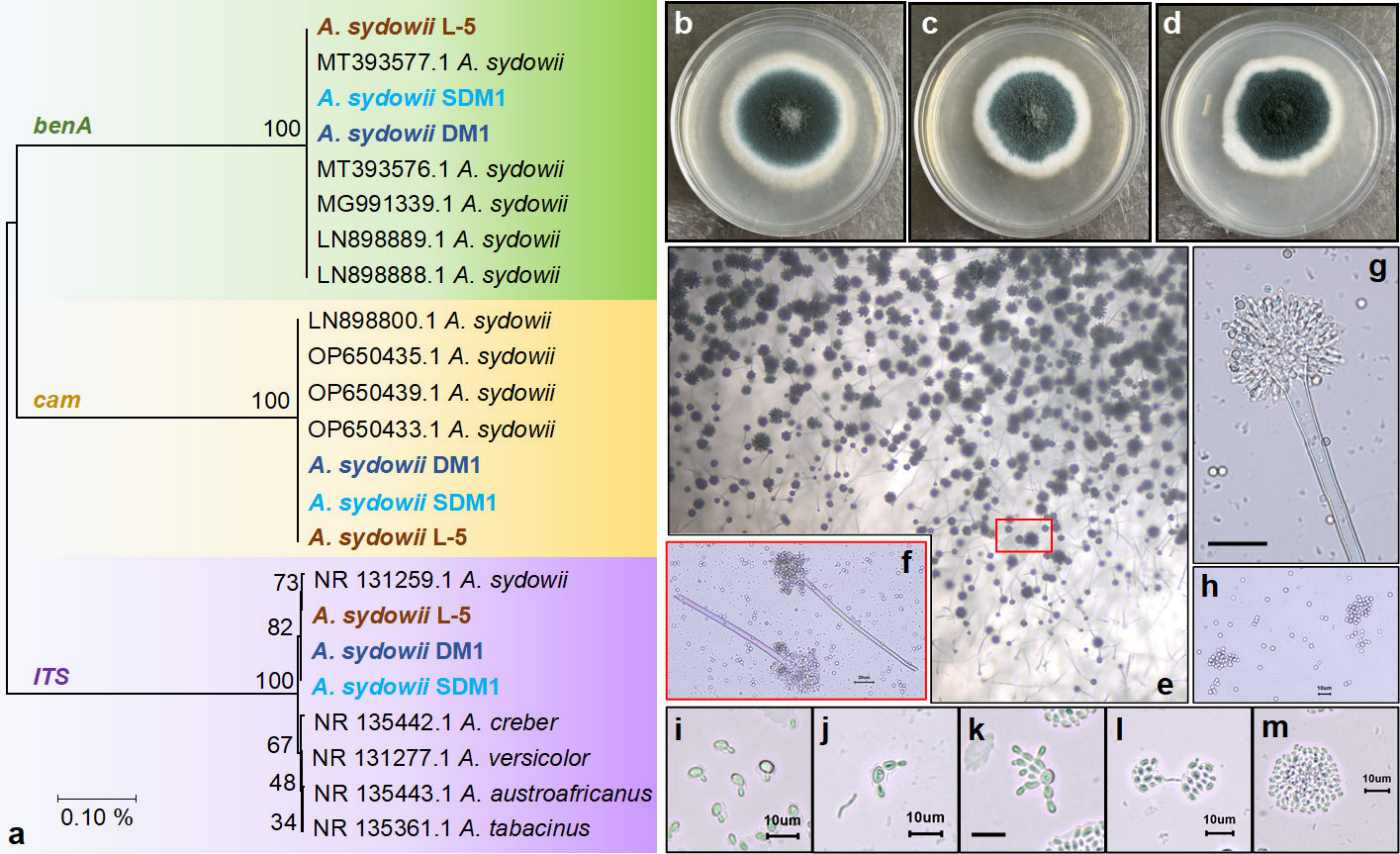
Identification of *A. sydowii* from different sources. (a) Phylogenetic tree of ITS, *benA,* and *cam* of three *A. sydowii*. Three *A. sydowii* owned the most closet relative to *A. sydowii* CBS 593.65 (NR 131259.1) under ITS amplification, and all obtained nucleotide sequences of these homogeneous strains were highly similar: 100% identity in *benA* and *cam*. Bootstrap analysis was performed with 1,000 replicates. (**b–d**) Plate morphology of *A. sydowii* originating from hadal, shallow, and terrestrial areas, respectively, on PDA after 7 days of culture at 28°C. (e) Morphological characterization of *A. sydowii* DM1 grown on PDA directly observed by microscope after 7 days of culture at 28°C. (**f–h**) Conidiophores and conidia on PDA. (**i–m**) Process of conidial germination at continuous timeframes (add 3 hours each from left to right). Scale bar represents 10 µm.

In our study, a hadal fungus, *A. sydowii* DM1, was isolated from Mariana Trench sediments of 10,898 meters and identified based on morphological identification and ITS sequence. To distinguish the piezotelerance of *A. sydowii* from different depths, we obtained two homogeneous fungi from different niches. One of *A. sydowii* strains from the shallow sea was named as SDM1 and the other *A. sydowii* was isolated from mud samples of Luchaogang Beach named as L-5. The phylogenetic tree was constructed by the conserved sequence of ITS, *cam*, and *benA*, which suggested all isolates belonged to *A. sydowii* (Fig. 1a).

The morphological colony of the three *A. sydowii* on PDA after 7 days of culture at 28°C was almost similar with atrovirens ascus and white margin (Fig. 1b through d). The micromorphology of *A. sydowii* DM1 was further observed, including the phenotype of conidiophores and conidia and the process of conidial germination at continuous timeframes. Conidiophores of *A. sydowii* DM1 appeared dark green (Fig. 1e) and characterized as typical columnar conical heads with the phialides (Fig. 1f and g). Bunchy germination tubes were observed within 12 hours (Fig. 1i through l), and after 15 hours, clusters of germinated conidium were formed (Fig. 1m). We also evaluated the salt tolerance of *A. sydowii* by measuring growth diameters on PDA at different NaCl concentrations (Fig. S2), which turned out the 0.5 M NaCl was the optimal concentration and there was an apparent tardiness growth rate under over 1.5 M NaCl in PDA.

### Spores of *A. sydowii* DM1 owns better effective metabolic activity after HHP treatment

The ability of spores to break dormant is one of the symbols for their environmental tolerance. In order to estimate the metabolic activity of spores after being treated with HHP, spores of *A. sydowii* were exposed to the elevated HHP, and their metabolic activities were tested using the resazurin. The heatmap was drawn with the normalized fluorescence value at different times.

**Fig 2:**
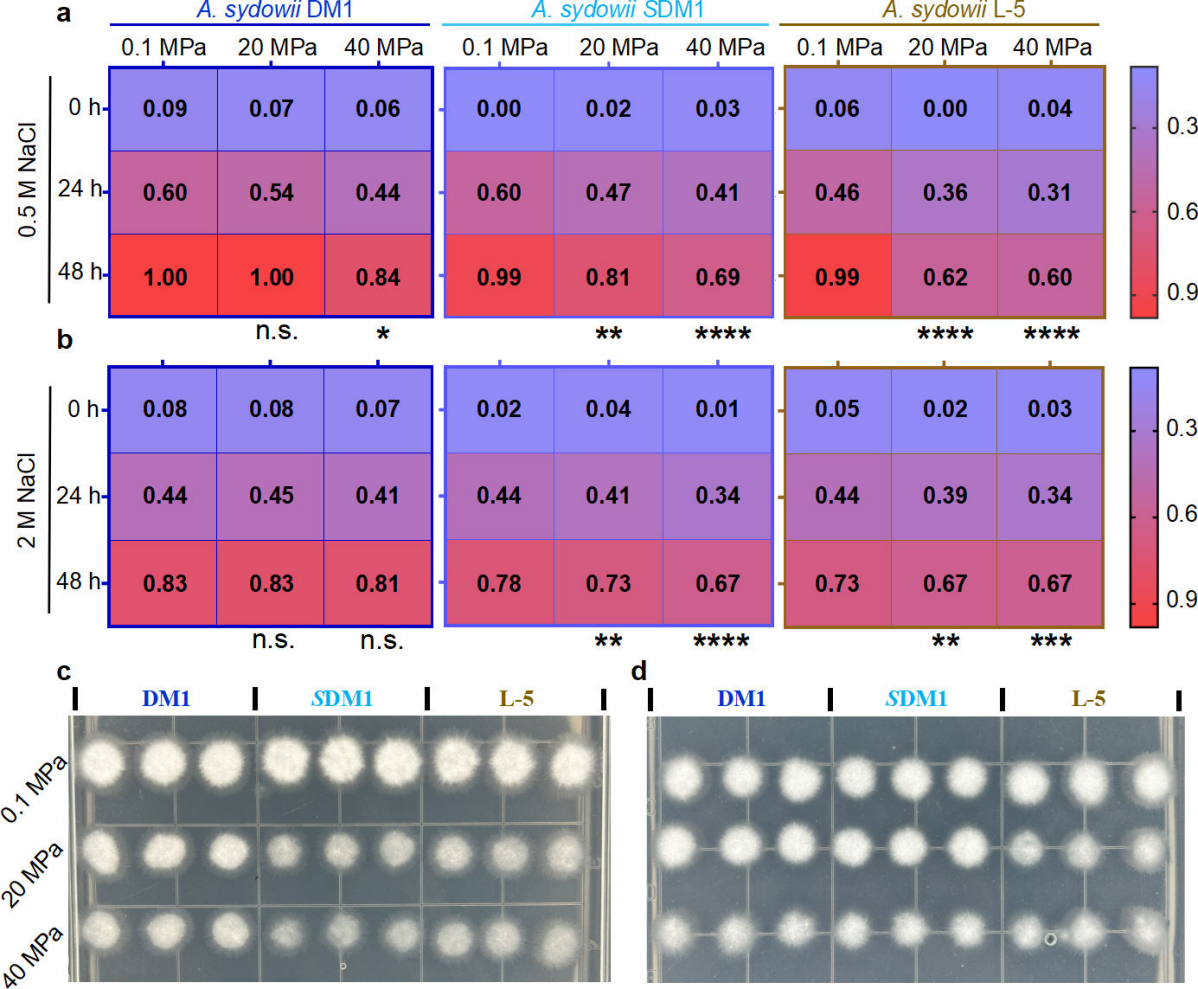
Metabolic activity of HHP-treated spores of *A. sydowii* indicated by resazurin test. (a) Heat maps showed the metabolic activity of three *A. sydowii* spores inoculated in minimal medium with 0.5 M NaCl for incubation at 37°C after HHP treatment. (b) Heat maps showed the metabolic activity of three *A. sydowii* spores inoculated in minimal medium with 2 M NaCl for incubation at 37°C after HHP treatment. The data are the mean of three replicates. Significance analysis was based on fluorescence values at 48 hours, and the atmospheric fluorescence values of each species were used as controls. Asterisks represent statistically significant differences determined by one-way ANOVA (n.s., *P* > 0.05, *P < 0.1, **P < 0.01, ***P < 0.001, and ****P < 0.0001). (c, d) Direct observation of spores from different *A. sydowii* cultured on PDA to compare their growth after elevated HHP. Specifically, 2.5-µL spores from two salinities were added on PDA after elevated HHP stimulation.

The results showed that only *A. sydowii* DM1 in 20 MPa could recover within 48 hours (fluorescence value was the same as control) (Fig. 2a). It was pronounced to *A. sydowii* L-5 as HHP surged, and the fluorescence value was lagged (0.60 in 40 MPa vs 0.99 in 0.1 MPa) (Fig. 2a). While the fluorescence value of *A. sydowii* SDM1 in elevated HHP chased closely with the one under 0.1 MPa, there were still significant differences compared with the control (0.81 in 20 MPa vs 0.99 in 0.1 MPa). Next, we added 2.5 µL HHP-treated spore suspensions to PDA to observe its revitalization. The results showed that the growth rates of *A. sydowii* SDM1 and L-5 at 20 and 40 MPa, respectively, were visually slower than that of ambient pressure (only light and thin mycelium) (Fig. 2c), while the development of *A. sydowii* DM1 was more mature compared with *A. sydowii* SDM1 and L-5 at elevated HHP. Those results demonstrated that hadal-derived fungi possess greater metabolic activity under elevated HHP compared other strains originating from the non-deep-sea environments.

Based on curiosity about the interaction between salinity and HHP for microbes in deep sea, we performed the same experiments to analyze the metabolic activity under both hypersaline (2.0 M NaCl) and high-pressure conditions. Interestingly, under 40 MPa, statistical significances of fluorescence values of three *A. sydowii* spores under hypersaline compared to that of 0.5 M NaCl were decreased, which demonstrated the metabolic activity of spores under dual stress seemed to have improved, especially for *A. sydowii* L-5 (Fig. 2b). We also carried out the above colony determination on PDA, and growth conditions under 20 MPa gained a well promotion (Fig. 2d). This result implied that the metabolic capability of spores may be enhanced under hypersaline conditions.

### Polarized growth conidia of *A. sydowii* DM1 grows faster under elevated hydrostatic pressure

**Fig 3:**
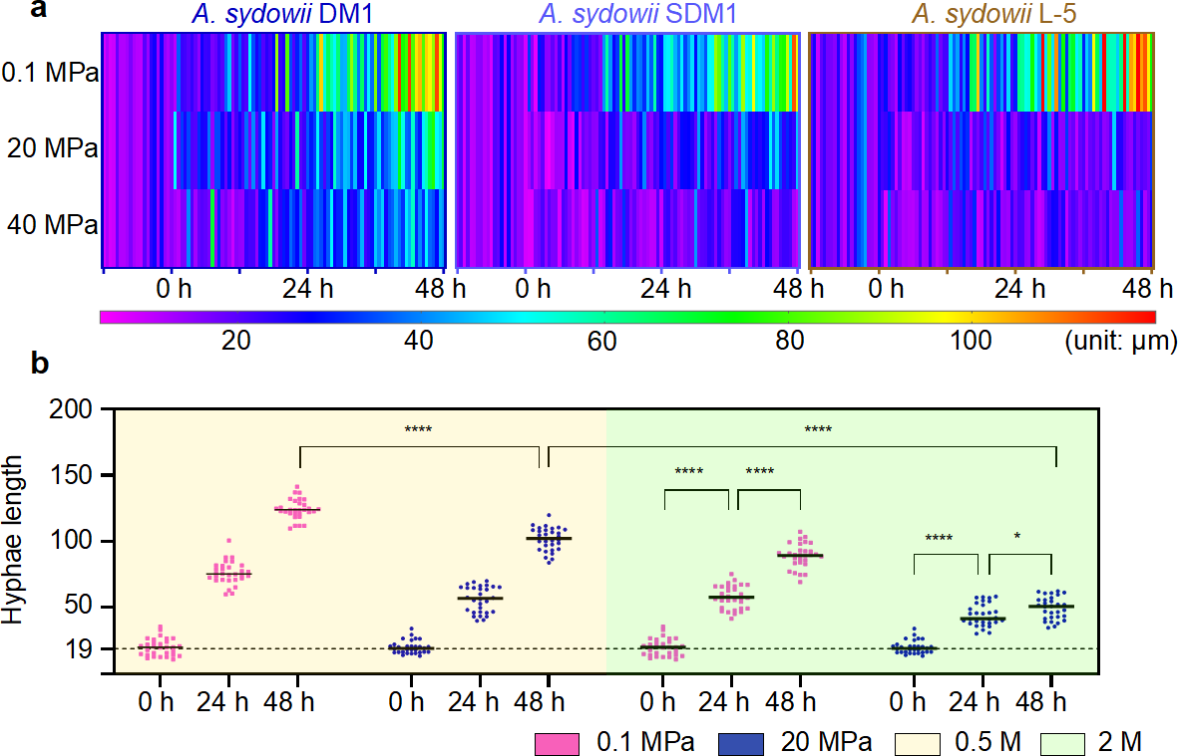
Hyphal length growth rate of polarized growth conidia of *A. sydowii* under elevated HHP. (a) *A. sydowii* from different sources grown on PDB (0.5 M NaCl) under elevated HHP. (b) Growth rate of *A. sydowii* DM1 on PDB under hypersaline/HHP (0.5 M vs 2 M NaCl; 0.1 MPa vs 20 MPa) conditions. Results indicated that *A. sydowii* DM1 showed growth defects under both hypersaline and HHP conditions. On the contrary, the polarized growth conidia grew rapidly within 48 hours under sole stress (hypersaline or HHP). The data are the mean of 25 replicates. *n* = 25 biologically independent samples. Asterisks represent statistically significant differences determined by one-way ANOVA (*P < 0.1 and ****P < 0.0001).

For the purpose of evaluating the growth and development of hyphae under HHP, we adopted a method of hyphae length measurement. The polarized growth conidia (20 µm) of three *A. sydowii* were measured every 12 hours under elevated HHP, and then, heatmaps were drawn to observe the tendency of hyphae growth rate (Fig. 3a). In our results, *A. sydowii* L-5 lost the ability of hyphal elongation under any HHP conditions, while *A. sydowii* SDM1, derived from shallow water, was able to grow under 20 MPa. *A. sydowii* DM1 was the only fungus that could grow slowly under 40 MPa, and the growth rate under 20 MPa was consistent with that of ambient pressure within 24 hours (Fig. 3a). Therefore, the results suggest that the capacity for fungal growth in HHP strengthens as the vertical depth of origins deepens, and *A. sydowii* DM1 owns well piezotolerant features which contribute to maintaining growth in elevated HHP.

To explore the interaction between HHP and other extreme conditions, we detected the development of polarized growth conidia of *A. sydowii* DM1 under both elevated HHP and hypersaline conditions. Our results showed that growth of *A. sydowii* DM1 seemed to be stunted by both hypersaline and HHP conditions since there was almost no distinct growth in 24–48 hours (Fig. 3b). Those indicate that it would be a disaster for *A. sydowii* DM1 to grow under double extreme conditions.

### Morphological switch under elevated hydrostatic pressure

**Fig 4:**
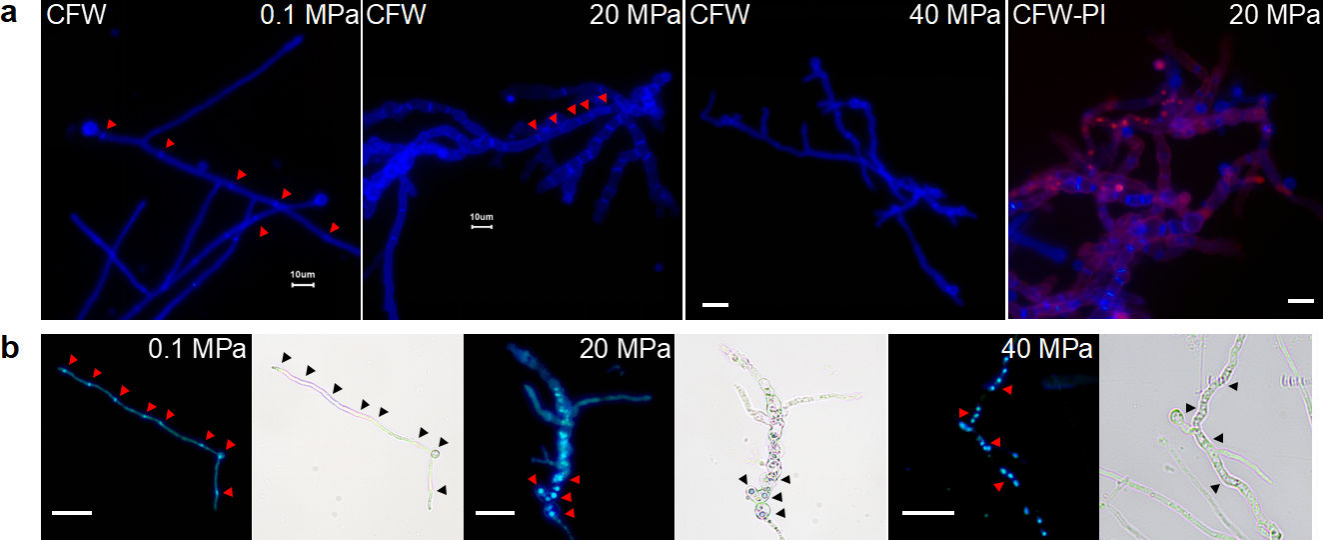
HHP-treated hyphae stained by fluorochrome. (a) Hyphae of *A. sydowii* DM1 stained with CFW and PI under elevated HHP. (b) Hyphae of *A. sydowii* DM1 stained with DAPI. The red arrow pointed to the septa or nucleus stained with fluorochrome. Scale bar represents 10 µm.

The micro-morphological characters of *A. sydowii* from all three origins at 0.1, 20, and 40 MPa were examined after incubation for 7 days at 28°C (Fig. 4). We captured at least 10 images using microscopy and conducted three repetitions for every sample. The results showed that 100% hyphae of *A. sydowii* DM1 under 20 MPa exhibited large vacuoles in every compartment with multiple quantities; however, hyphae under 40 MPa showed no swelling except obvious runty, dented, and curling cells (Fig. 4a). The similar hypha structure was also observed in the other two *A. sydowii*, despite they visually appeared to be quite short and incomplete (Fig. S3a and b). Interestingly, the dual-staining of hyphae with CFW-PI was clearly displayed in 20 MPa (Fig. 4a), indicating that the swollen one could be penetrated by PI dye, whereas only a small part of the hyphae under 40 MPa was stained by PI (Fig. S3c). Microscopic examination also found that hyphal nucleus appeared orderly in line under ambient conditions, while the nucleus formation began to become irregular and piled up together in chaos under elevated HHP (Fig. 4b). These observations clearly suggested that HHP affects hyphae extension and development and integrity of the cell wall.

### Transcriptome overview, differentially expressed genes, and enrichment analysis

To understand the fungal piezotolerance at a molecular level, we performed a transcriptome analysis based on RNA sequencing of *A. sydowii* DM1 under various HHP conditions. Raw reads information was listed in Table S2, which indicated the reliability of RNA-Seq data in this work. Besides, overall transcription levels were quantified by FPKM metrics. We then analyzed the global gene differences between the groups (Fig. S4). The principal component analysis of the gene expression profiles suggested that expression profiles of *A. sydowii* DM1 under elevated HHP emerged distinguishingly between individual groups. Besides, a total of 2,410 and 5,176 DEGs were detected in *A. sydowii* DM1 cultured under 20 MPa and 40 MPa, respectively (Fig. S4b and c). In these DEGs, commonly shared genes (a total of 1,530 genes) were enriched in Fig. S5, which might imply their potential functional role under HHP and need to be analyzed further.

**Fig 5:**
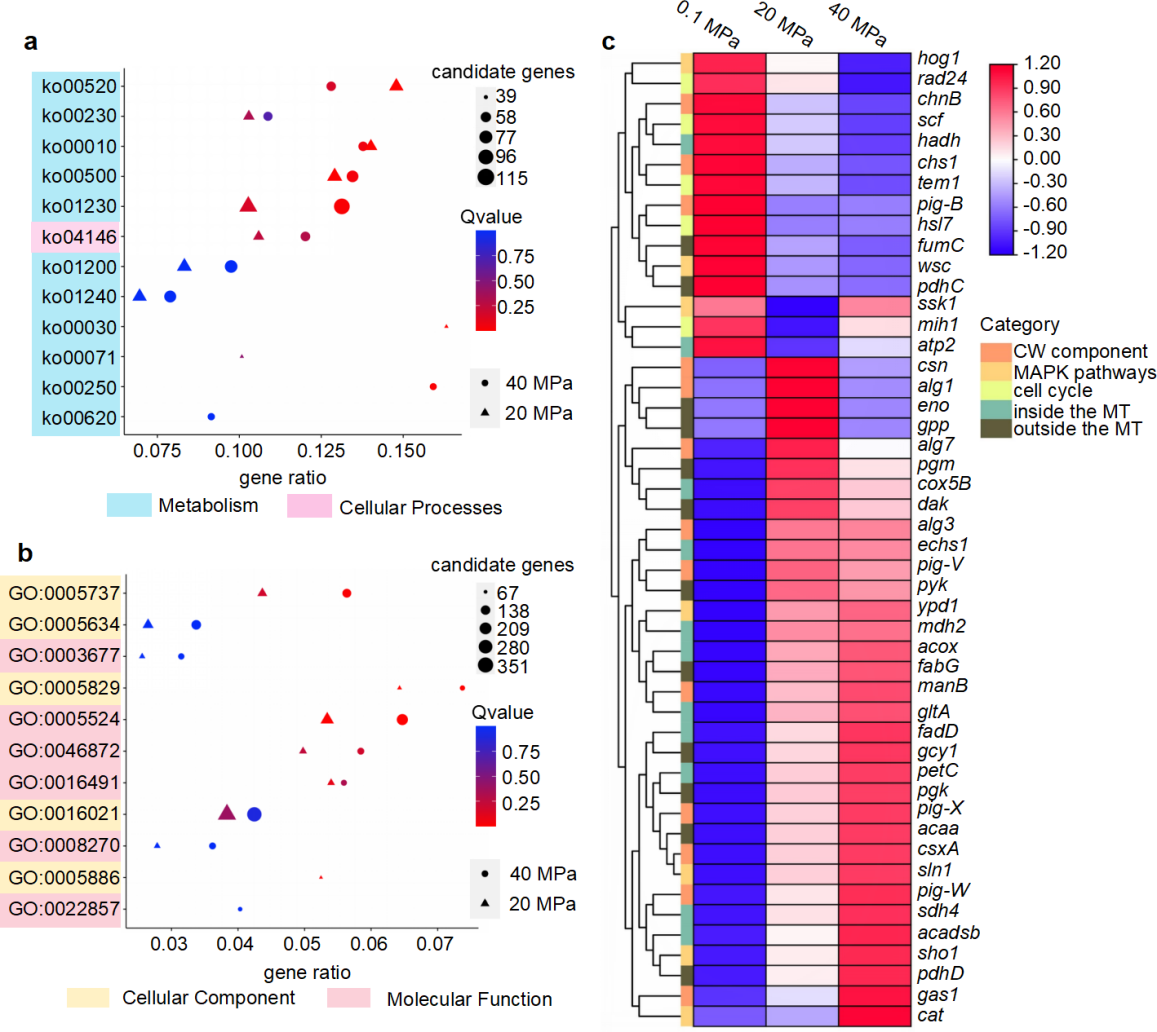
DEG overview of *A. sydowii* DM1 cultured under elevated HHP for 3 days. (**a, b**) Top 10 terms in KEGG (**a**) and GO (**b**) enriched from DEGs of the 20-MPa and 40-MPa groups (log_2_ fold-change > 1.5 or <−1.5). Among of them, biosynthesis of amino acids (ko01230; 90 candidate genes in 20 MPa and 115 candidate genes in 40 MPa) was occupied mostly in KEGG enrichments, including carbon metabolism (ko01200), starch and sucrose metabolism (ko00500), and peroxisome metabolism (ko04146). In the top terms of GO enrichments, there were two main functional categories: cellular component and molecular function. The integral component of membrane (GO:0016021) occupied the majority with 317 candidate genes in 20 MPa and 351 candidate genes in 40 MPa. (c) Normalized FPKM average value of marker genes analyzed in this study. CW, cell wall; MT, mitochondrial.

We enriched all DEGs in KEGG and GO (Fig. S6) and further enriched the top 10 items from all groups in Fig. 5; Table S4. These results implied that biosynthesis of amino acids, carbohydrate metabolism (CarM), and cell membrane play an essential role in confronting the HHP condition. Next, TBtools (v1.120) was used to draw a heatmap containing the main marker genes related to an essential role in HHP resistance (Fig. 5c). These genes were attributed to environmental stress and intracellular metabolism, including the cell wall component, MAPK pathways, cell cycle, and CarM, which could potentially enhance the pressure tolerance of *A. sydowii* DM1.

To further verify the RNA-Seq data, RT-qPCR was applied to test the relative expression levels of seven key DEGs (Fig. S7). Results indicated the reliability of RNA-Seq data and further demonstrated that transcriptome was a productive technique for studying the intracellular mechanism of filamentous fungi under HHP.

### Cell wall composition shows variations under elevated hydrostatic pressure

**Fig 6:**
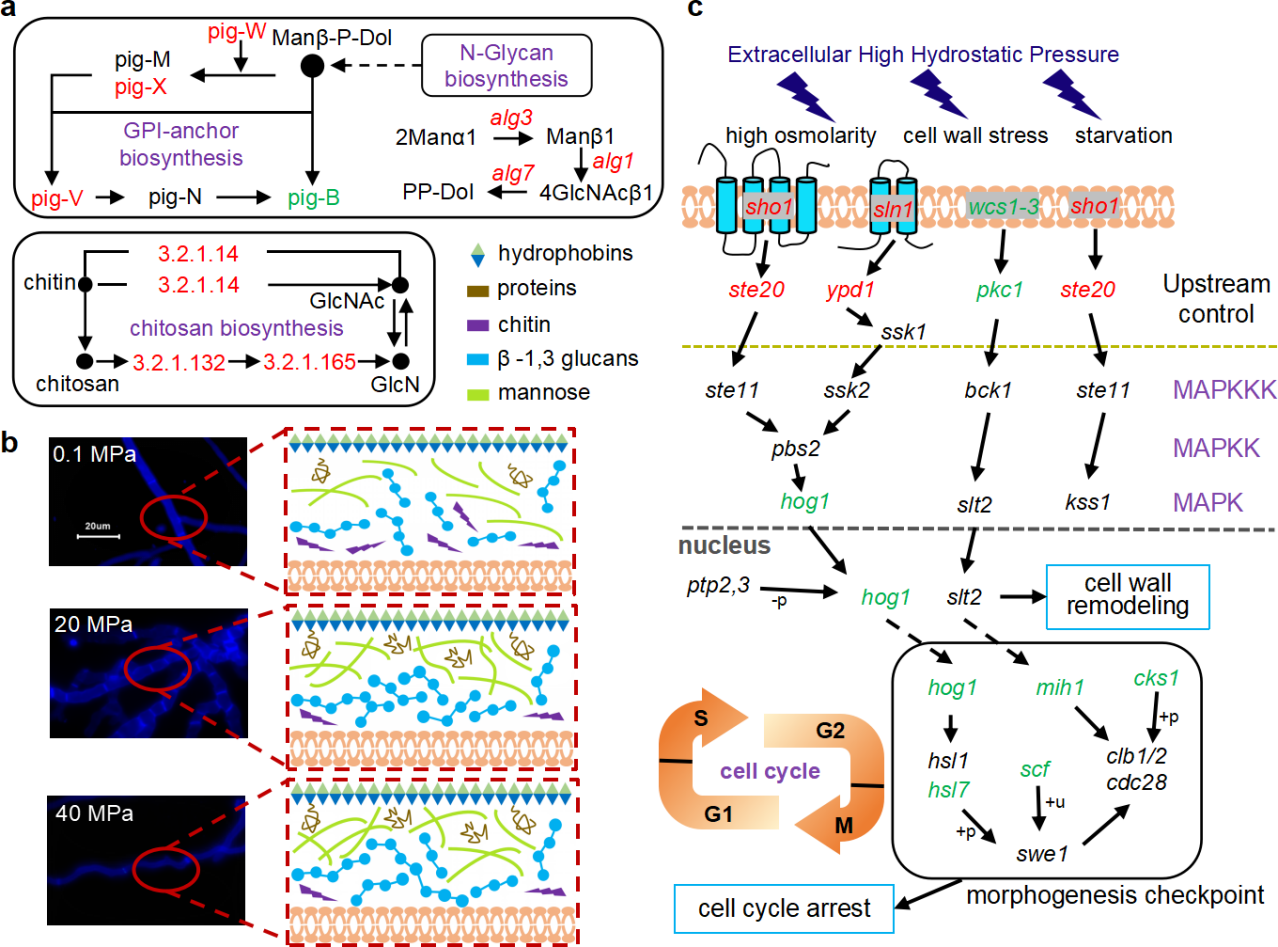
Pathways of phenotypic modulation analyzed by the overview of DEGs. (a) Main cell wall component biosynthesis pathways. (b) *A. sydowii* DM1 conceptual cell wall components under elevated HHP analyzed by transcriptome. (c) DEGs from MAPK signaling pathways to cell cycle in *A. sydowii* DM1 under elevated HHP. Red, green, and black boxes/letters indicate genes upregulated, downregulated, and not significantly expressed, respectively.

In our results, hydrostatic pressure induced major changes in the transcriptional levels of DEGs related to cell wall components. Proteins usually are anchored on the outer layer by glycosylphosphatidylinositol, which play roles in maintaining cell wall architecture and interacting with host cells in yeasts and filamentous fungi (45). Here, we detected upregulation of related genes, such as *pig-W*, *pig-X*, *alg1* coding for β−1,4-mannosyltransferase, and *man2c1* coding for alpha-mannosidase (Fig. 6a; Table S3). These enzymes coordinate the assembly of the lipid-linked oligosaccharide precursor (Glc3Man9-GlcNAc2), which contributes to the formation of mannoproteins (46). Another key gene *gas1*, which is responsible for the prolongation of β-1, 3-glucan, was increased (Table S3). Meanwhile, we compared the genes related to chitin synthase in the GO database, and most of them were downregulated (Table S3). We also found that genes associated with the biosynthesis of chitinase, which attacks the crystalline structure of chitin by fracturing the glycoside saccharide bond. Finally, we proposed the main shifts of cell wall composition under elevated HHP in Fig. 6b and speculated that the cell wall structure with increased glucans and lipid-anchored proteins improved the piezotolerance of *A. sydowii* DM1.

In fungi, the septum is initially formed by a series of accumulations of actin, which recruits *myo-2* to produce contractile force; then, protein *bud4* gathers *bud3* and *rho-4* to determine the exact location for contractile actomyosin ring (CAR) formation, and finally, the cell wall is constructed (47, 48). Here, we found the septum formation-related genes were differently regulated under elevated HHP (downregulated in 20 MPa while not significantly expressed in 40 MPa; Table S3), including myosin complex, rho family (a signaling protein that presumably affects septins, formins, and p21-activated kinases), and gene *mih1* (a previous report showed that a shorter distance between the septa was observed in *mih1* mutants [49]). Consequently, we assumed that under 20 MPa, myosin proteins and rho complex were not yet activated to find the exact location for CAR formation; then, they randomly distributed within the hypha and directly contracted; as a result, irregular and growing numbers of septa were formed (Fig. 4).

### High-osmolarity glycerol pathway responses to hydrostatic pressure by regulating cell cycle arrest

The HOG-MAPK signaling pathway has been well studied in many *Aspergillus* species in response to a series of environmental stresses including osmotic, oxidative, and heat (50, 51). In this work, the expression of genes *sln1* and *sho1* was more activated with the higher pressure; other genes such as *wcs1-3*, *pkc1*, and *hog1* were downregulated (Table S3; Fig. 6c). Generally, *sho1* and *sln1* both control the HOG-MAPK with negative feedback regulation (52). The upregulated genes, *sho1* and *sln1*, with the help of the activation of upstream control gene *ste20* and *ypd1*, recognized as an activator of HOG-MAPK, led to the decreased transcriptional level of *hog1* (Fig. 6c).

Environmental stress induces cell-cycle delays, which announce cells to adapt to the stress before progressing into vulnerable cell cycle transitions (53, 54). Here, we found that the morphogenesis checkpoint regulated by *hog1* was the dominant “control” part, which made the G2/M phase of the cell cycle unable to proceed normally. Besides, gene *mih1* was downregulated, a gene that encoded a dual-specificity phosphatase and involved in the G2/M transition of the mitotic cell cycle by regulating the Cdk1 activity in eukaryotes (49). We also detected another dramatically reduced expression of *hsl7*, a protein that binds to *swe1. hsl7* interacted with *hog1* which led to the cell cycle arrest through the morphogenesis checkpoint (Fig. 6c). In conclusion, the activation of the HOG-MAPK signaling pathway might give cells more time to buffer against the external HHP and we also speculate that it is the main reason for the poor growth of *A. sydowii* DM1 under elevated HHP as the expression of *hog1* dropped sharply under 40 MPa (Fig. 4; Table S3).

### Activated carbohydrate metabolism enhances piezotolerance of *A. sydowii* DM1

**Fig 7:**
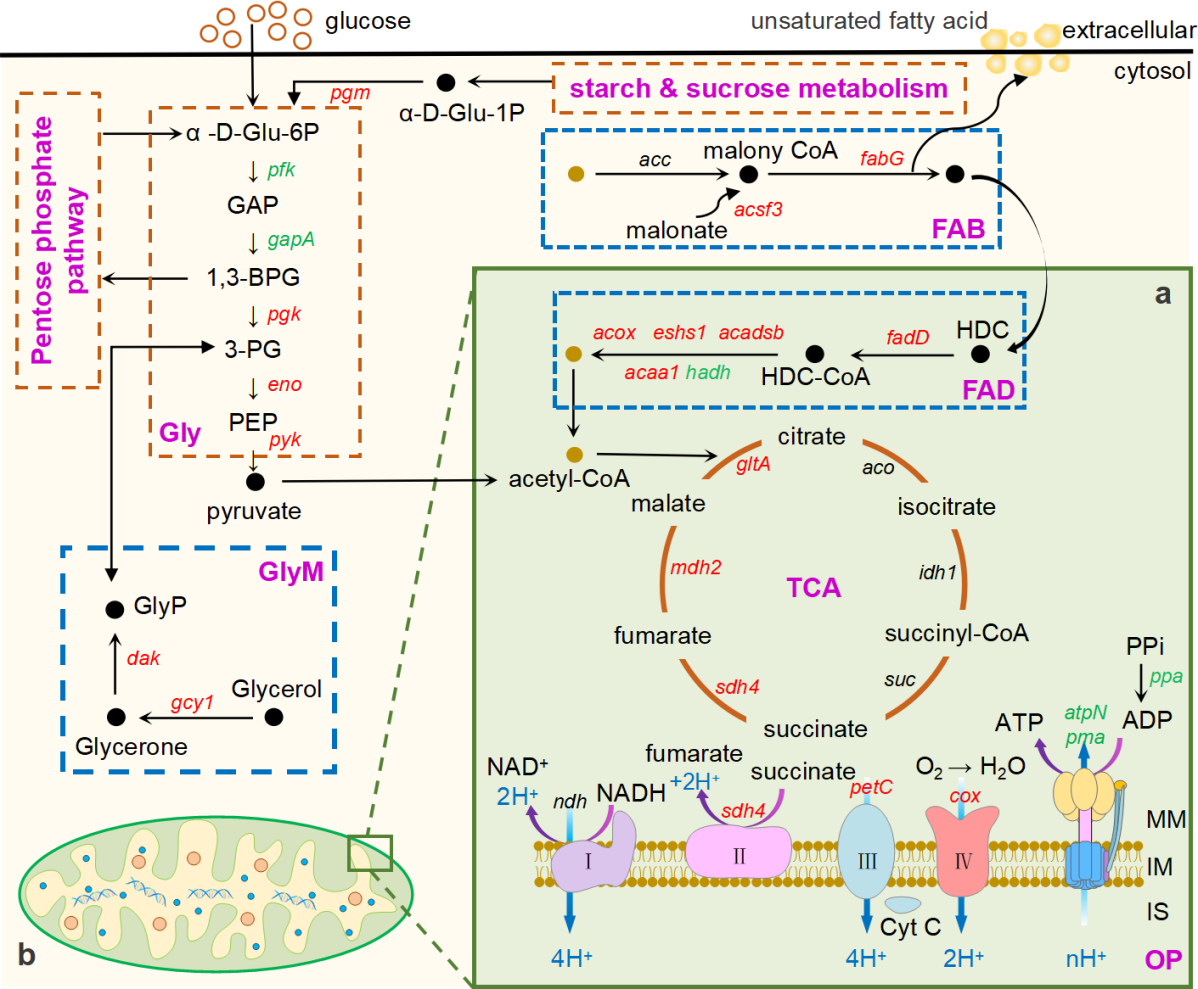
Carbohydrate metabolism of *A. sydowii* DM1 switched under elevated HHP. (a) Pathways related to elevated HHP in the mitochondria (light-green base). (b) Pathways related to elevated HHP outside the mitochondria (light-orange base). α-D-Glu-6P, α-D-Glucose-6P; α-D-Glu-1P, α-D-Glucose-1P; GAP, glyceraldehyde-3P; 1,3-BPG, 1,3-bisphosphoglycerate; 3-PG, glycerate-3P; PEP, phosphoenolpyruvate; HDC-CoA, hexadecanoyl-CoA; HDC, hexadecanoate; GlyP, glyceronephosphate; Gly, glycolysis; GlyM, glycerolipid metabolism; TCA, tricarboxylic acid cycle; OP, oxidative phosphorylation; FAD, fatty acid degradation; FAB, fatty acid biosynthesis; MM, mitochondrial matrix; IM, inner membrane; IS, intermembrane space.

Next, we noticed one of the most abundant enrichments—CarM—which also plays a potential role in stress tolerance, including heat stress, polycyclic aromatic hydrocarbon degradation, and piezotolerance (43, 55, 56). Then, we confirmed that the numbers of upregulated DEGs in CarM were double than those of downregulated ones (Fig. S9); hence, the associations were found between the pathways in the CarM. In the mitochondria matrix (MM; green box in Fig. 7), *sdh4*, *petC*, and *cox* belonging to the electron transport chain enzyme complexes were upregulated, while *pma* coding for H^+^ transporting ATPase was downregulated. Besides, genes related to the tricarboxylic acid cycle (TCA) and fatty acid degradation (FAD) were upregulated (Fig. 7a). In the cytosol, genes related to glycerolipid, fatty acid biosynthesis (FAB), and glycolysis were upregulated (Fig. 7b).

In our study, the negative expression of *pma* and *atpN* in MM caused the turn-off of the proton channel in ATP synthase, possibly leading to the transformation electrochemistry into heat energy (Fig. 7a). We found that the essence of this mechanism was similar with the uncoupler in oxidative phosphorylation, which would produce heat energy to liberate the electrochemical energy in the ATP synthase. Uncoupler controls energy expenditure and catabolism, such as carbohydrate, lipid, and protein (57). Furthermore, *pyk*, one of the limit-rating enzymes inducing the last step of glycolysis promoted the accumulation of pyruvate and aerobically turned into acetyl-CoA (Fig. 7b). With the help of citrate synthase *gltA*, acetyl-CoA could enter and activate the TCA. We believed that activation of the citrate cycle has two main functions: firstly, it re-generates energy currency ATP by substrate phosphorylation due to the respiratory chain inhibition; secondly, the intermediates produced in the TCA pathway will serve as precursors for HHP tolerance. For example, genes related to amino acid synthesis are upregulated, such as *gad1*, *gdh2*, and *argG* (Table S3), leading to the formation of 2-ketoglutarate and oxaloacetate, which in turn act as the starting points for the synthesis of glutamate and aspartate, respectively.

FAD, another pathway that generates acetyl-CoA, was also observed. A long-chain acyl-CoA synthetase *fadD* was one of the critical synthetases for activating the initial step of FAD (Fig. 7a); the upregulation of this gene continuously enhanced the catalytic ability in each cycle, thereby releasing more acetyl-CoA and FADH_2_ to enter TCA. Besides, acetyl-CoA acyltransferase encoded by *acaa1* was upregulated, which is a rate-limiting enzyme belonging to peroxisomal β-oxidation in the last step of DHA biosynthesis. Although we did not detect significant upregulation of *acaca* (a gene coding for acetyl-CoA carboxylase), we detected upregulation of *acsf3* coding for malonyl-CoA synthetase, which would prime the initial step of FAB. The acetyl-CoA oxidoreductase *fabG* was also upregulated, which together would synthesize unsaturated fatty acids and intensify cell membrane fluidity (Table S3). In conclusion, our results demonstrated that *A. sydowii* DM1 resisted elevated HHP by maintaining the cell membrane fluidity and permeability.

### Three *A. sydowii* showed different gene expression levels under elevated HHP

In order to assess the extent of HHP adaptation in different isolates, we further examined gene expressions of both *A. sydowii* SDM1 and L-5 under the same HHP conditions as *A. sydowii* DM1. Marker genes related to the HOG-MAPK, carbohydrate metabolism, and synthesis of unsaturated fatty acids and cell walls were selected, and their expression was tested by RT-qPCR. Our results showed that the expression of marker genes involved in HHP adaptation varied between these three isolates (Fig. S8). For instance, at pressures under 20 MPa, partial genes showed the opposite expression pattern, including *hogA* and *gas1*. It was worth noting that under 40 MPa, the alterations in expression levels of marker genes in *A. sydowii* DM1 were more pronounced than those in the other two strains (Fig. S8). This was the case for genes including *hogA*, *gas1*, and *fadD*, indicating that the metabolism of *A. sydowii* DM1 was relatively active. The results were consistent with our examination of hypha growth, demonstrating that only *A. sydowii* DM1 had a significant growth trend at pressures below 40 MPa (Fig. 3a). In summary, the study revealed an upregulation of genes related to the synthesis of unsaturated fatty acids and carbohydrate metabolism, while the detailed piezotolerant mechanisms of different sources of *A. sydowii* may differ. Furthermore, the higher level of gene expression detected under HHP suggests that *A. sydowii* DM1 from the hadal area possesses unique metabolic mechanisms, although further study is needed.

## DISCUSSION

More and more deep-sea fungi and their potential roles, such as the ecological functions and novel secondary metabolites productions (3, 9, 58), were discovered from various depths in the deep sea. However, apart from some prokaryotes and yeast species (10, 13, 59), limited information is available for adaptation and genome informatics of culturable filamentous fungi under HHP, and relatively few studies paid attention to the relationship between their physiological property and HHP (13, 14, 17). In our work, we used three homogeneous *A. sydowii* as the representative to explore the piezotolerance of filamentous fungi. The same species with different origins (terrestrial, shallow, and hadal area) allow us to better understand how filamentous fungi respond to HHP. As far as we know, similar studies involving two or more similar filamentous fungi from different ecological niches have never been documented. Furthermore, we evaluated their piezotolerance via two aspects: growth rate and morphological changes. Transcriptome was further used to explore the internal mechanism of *A. sydowii* DM1 under HHP. This should be the most comprehensive study of filamentous fungi growth under elevated HHP in recent years and sheds light on the future study in both piezosensitive and piezophilic mechanisms, which is marked as an inspiring achievement.

Early studies on culturable filamentous fungi, to our knowledge, were mostly under mild HHP (17, 60), and very few of them exceed 30 MPa (9, 18, 32). Considering the truth that conidia and hyphae showed serious growth defects under 60 MPa in our preliminary experiment, a compromised pressure of up to 40 MPa was ultimately employed in our study. Although there’s much more to investigate further, that is a valuable leap for our understanding of hadal fungi. In addition, for halophilic ascomycetous model *A. sydowii*, hyperosmotic conditions (2.0 M NaCl) induce the transcriptional cell wall reconstruction, hydrophobin production, and glycerol synthesis (61), while such regulatory mechanisms were not fully observed at an optimal salt concentration (0.5 M NaCl) (62). These two gradients are suitable for exploring the different intrinsic relationships between HHP and osmotic conditions. On the other hand, *A. sydowii* is well known as a pathogen of corals (63), which is ubiquitously distributed in every corner of the earth, including soil (64), dried food (65), waters of the salterns (66), extreme environments, such as deep-sea mud, and Antarctic region (67, 68). Notably, *A. sydowii* from different sources owns unique characteristics that demonstrate its adaptability to the living environment. For instance, terrestrial isolates were consistently non-pathogenic for corals (69, 70), whereas deep-sea isolates exhibited piezophilic adaptation (17). In our study, the results demonstrate that *A. sydowii* DM1, originating from the hadal area, exhibits superior piezotolerant ability and increased expression of genes associated with HHP tolerance mechanisms when compared with strains from other locations. (Fig. S8). Our results are similar with the report that Roumagnac M. et al. compared the growth and cell phenotypes under HHP of two homogeneous strains; the strain from the deep biosphere performed well in metabolism and formation of chained cells (71), which indicated that even microbes of the same species have different strategies for responding to HHP. In short, we believe the *A. sydowii* DM1 with distinctive piezotolerance mechanism is a valuable model filamentous fungus for HHP study in the perceivable future.

We performed a series of staining experiments to observe the variations of hyphae under elevated HHP. One of the findings was the significantly different phenotype of hyphae under 20 MPa, including increased septa, swollen hyphae, and penetration of PI dye (Fig. 4). PI dye is well known to penetrate the damaged cell membranes and stain only the inactivated or damaged cells (39, 72). However, in our study, we do not consider hyphae of *A. sydowii* DM1 under 20 MPa as an inactivation condition, since the growth rate of overall mycelium was faster than that under 40 MPa (Fig. 3a); hence, we believe that the hyphae alter the permeability of cell membrane under HHP. Besides, the function of a hyphal septum is still an open question. One of the ideas is that hyphae with increased septa could withstand turgor pressure by increasing stiffness (47). According to these two points, we provide proof that, for *A. sydowii* DM1, 20 MPa is an ambiguous condition where hyphae could regulate the number of septa to better maintain its swollen structure and the permeability of cell membrane to proactively survive, while HHP over 40 MPa is destructive to *A. sydowii* DM1 or even worse to those non-piezophillic microbes (*A. sydowii* SDM1 and L-5) that they passively tolerated this condition during their growth. We further analyzed the cell wall composition, which is an essential structure with great plasticity in fungi and can be dynamically remodeled due to changes in temperature and pH and oxidative and osmotic stresses (73–75). Abe F. found that genes of *S. cerevisiae-*encoded mannoprotein of the cell wall were upregulated under mild HHP (76). Consistent with our results, for *A. sydowii* DM1, we presumed that one of its deep-sea characteristics would be able to autonomously regulate the cell wall structure to withstand external HHP during their growth. Although the intrinsic mechanism of fungal cell walls in response to environmental stress remains unknown, we believed that the increased content of glucans and mannoproteins did provide a relatively stable environment inside the cell of *A. sydowii* DM1, but further verification is needed.

Other two interesting points were found in our transcriptomic data. One of them was that in *A. sydowii*, the expression patterns of the HOG system under HHP are analogous to that under hyperosmotic conditions. Previous studies had found that under hyperosmotic conditions, *A. sydowii* EXF-12860 would utilize *hog1* by accessing the mitogenic checkpoint to achieve cell cycle arrest which generated adaptive responses against external stress (77, 78). These double checkpoints controlled by *hogA* would be one of the reasons why mycelium barely grew under the dual-stress conditions (high salinity and high pressure). Another is *A. sydowii* DM1 exhibited poor growth under elevated HHP with an active metabolism. A similar study also reported that *Bacillus subtilis* cells can survive deep starvation conditions for months and appear to be metabolically active. Another example is *A. borkumensis*, showing a limited growth yield under elevated HHP while pathways related to energy generation were upregulated (30, 79). ATP will be produced throughout the whole process of active metabolism to provide energy for living organisms, and the mitochondrial oxidative respiratory chain is one of the key approaches for eukaryotic cells (80, 81). While yielding ATP is an energy-consuming process, we believed that to save more energy components to resist HHP, *A. sydowii* DM1 alternatively inhibited the respiratory chain and activated the TCA cycle for ATP production; as a result, more beneficial precursors or substances were obtained under elevated HHP with low energy consumption. However, a related study suggested that *V. parahaemolyticus* could use the energy transmitted by electrons to pump H^+^ out of the cell under HHP stress for the purpose of reducing intracellular H^+^ levels without consuming ATP (56). Given the potential impact of energy accumulation on cell growth and metabolism (82, 83), it is imperative that future research is conducted to investigate ATP accumulation within cells under HHP, which will provide a clearer understanding of the ability of *A. sydowii* DM1 to utilize ATP in the synthesis of stress-resistant substances.

**Fig 8:**
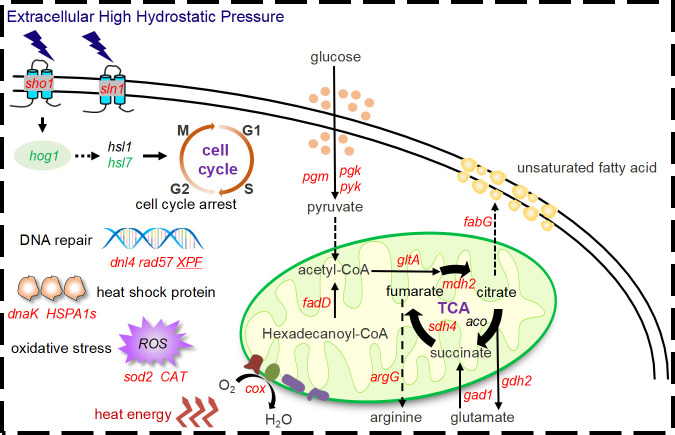
Schematic diagram of *A. sydowii* DM1 under elevated HHP.

Finally, we deduced the schematic diagram of piezotolerance in *A. sydowii* DM1 (Fig. 8). Firstly, the *hog1*-mediated HOG-MAPK pathway regulates cell cycle arrest which allows cells to have buffering time to adapt to the external HHP. Secondly, by activating carbohydrate metabolism, cells consume oxygen and produce heat during the activation of oxidative phosphorylation uncoupler. At the same time, the citrate cycle produces ATP through substrate phosphorylation, and the intermediate products of the citrate cycle are synthesized as precursors that can withstand HHP, such as unsaturated fatty acids, arginine, and glutamic acid. Last but not the least, other pressure-tolerance genes or proteins are also detected, such as DNA repair and heat shock proteins. To sum up, *A. sydowii* DM1 utilizes the above mechanisms to complete the tolerance and growth of environmental HHP.

Words, in the end, our works provided a novel insight for filamentous fungi to adapt to HHP conditions. Even though this article is just the tip of the iceberg for filamentous fungi under elevated HHP, it was an important step to understand their pressure adaptation mechanism. Here, in this article, we provided a relatively complete evaluation strategy on filamentous fungi under elevated HHP, and the molecular mechanisms by transcriptome were analyzed, which establishes a theoretical basis for the pressure tolerance and metabolic potential of deep-sea fungi in the future.

## Data Availability

The entire ITS, *benA*, and *cam* genes in all *A. sydowii* were submitted to the NCBI, accession numbers OR263168–OR263170 (ITS), OR345307–OR345309 (*cam*), and OR345310–OR345312 (*benA*). Raw sequencing reads for the transcript information in this work were submitted to the NCBI Sequence Read Archive under sequential accession numbers from SRR25302144 to SRR25302152. Correspondence and requests for materials should be addressed to Xi Yu.

## SUPPLEMENTAL MATERIAL

Supplemental Figures msystems.01085-23-s0001.docx Figures S1 to S9.

Tables S1 and S2 msystems.01085-23-s0002.docx Primers and statistical summary of the results of RNA-seq data.

Table S3 msystems.01085-23-s0003.docx Transcriptomic expression of *Aspergillus sydowii* DM1's genes.

Table S4 msystems.01085-23-s0004.docx Detailed information of top 10 terms in DEGs from all groups.

ASM does not own the copyrights to Supplemental Material that may be linked to, or accessed through, an article. The authors have granted ASM a non-exclusive, world-wide license to publish the Supplemental Material files. Please contact the corresponding author directly for reuse.
