# Supplementary material for: Insight into the adaptation mechanisms of high hydrostatic pressure in physiology and metabolism of hadal fungi from the deepest ocean sediment
Source: mSystems. 2023 Dec 20;9(1):e01085-23. doi: 10.1128/msystems.01085-23 (PMC10804941; doi:10.1128/msystems.01085-23)
Supplement: Supplemental Figures — Figures S1 to S9. [file msystems.01085-23-s0001.docx]

**Insight into the adaptation mechanisms of high hydrostatic pressure in physiology and metabolism of hadal fungi from the deepest ocean sediment**

Maosheng Zhong, Yongqi Li, Ludan Deng, Jiasong Fang, and Xi Yu^*^

**running head:** Hadal sediment fungi and HHP tolerance

**Address**: ^1^Shanghai Engineering Research Center of Hadal Science and Technology, College of Marine Sciences, Shanghai Ocean University, Shanghai, 201306, China

*Corresponding author: XY (x[yu@shou.edu.cn](mailto:yu@shou.edu.cn), +8615332036650);

**Key words:** Piezotolerance, hadal fungi, development, cell structure, metabolic activity

**Fig. S1**


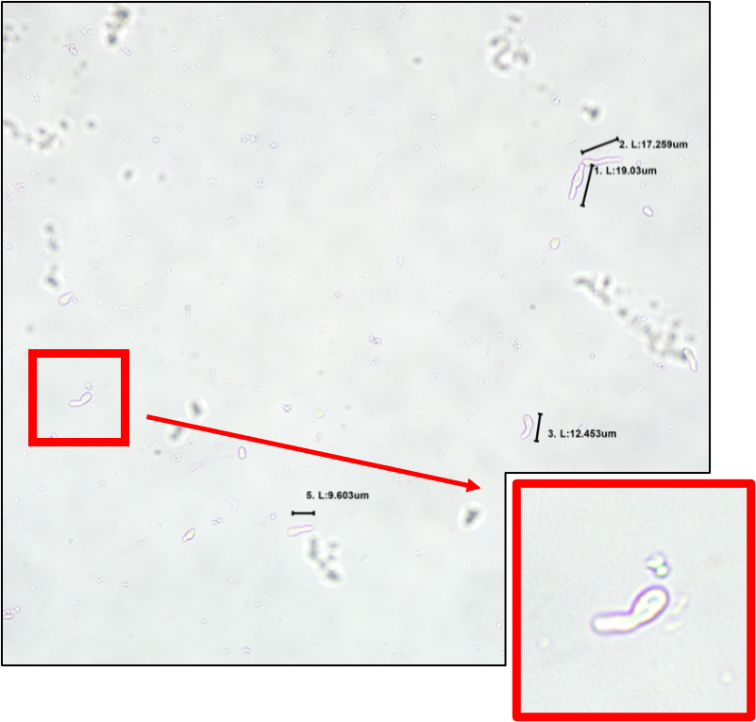


**Fig. S1** Polarized-conidia of *A. sydowii* were formed on PDB at 28 ºC after 12-16 h inoculation.

**Fig. S2**

**a**


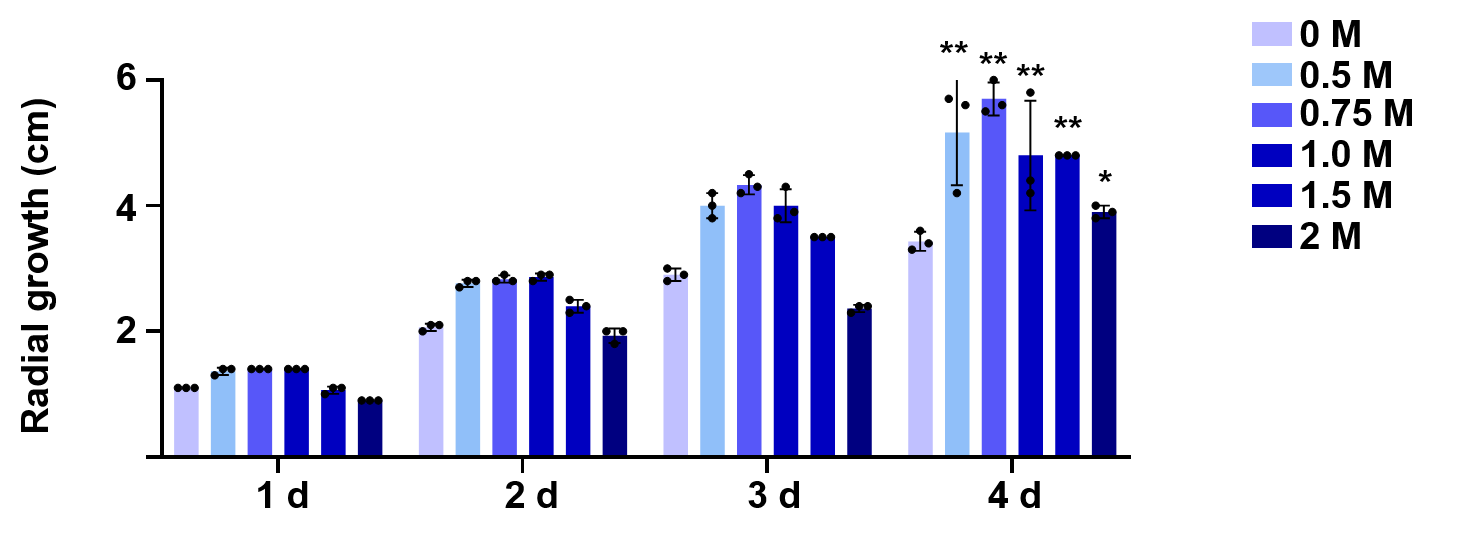

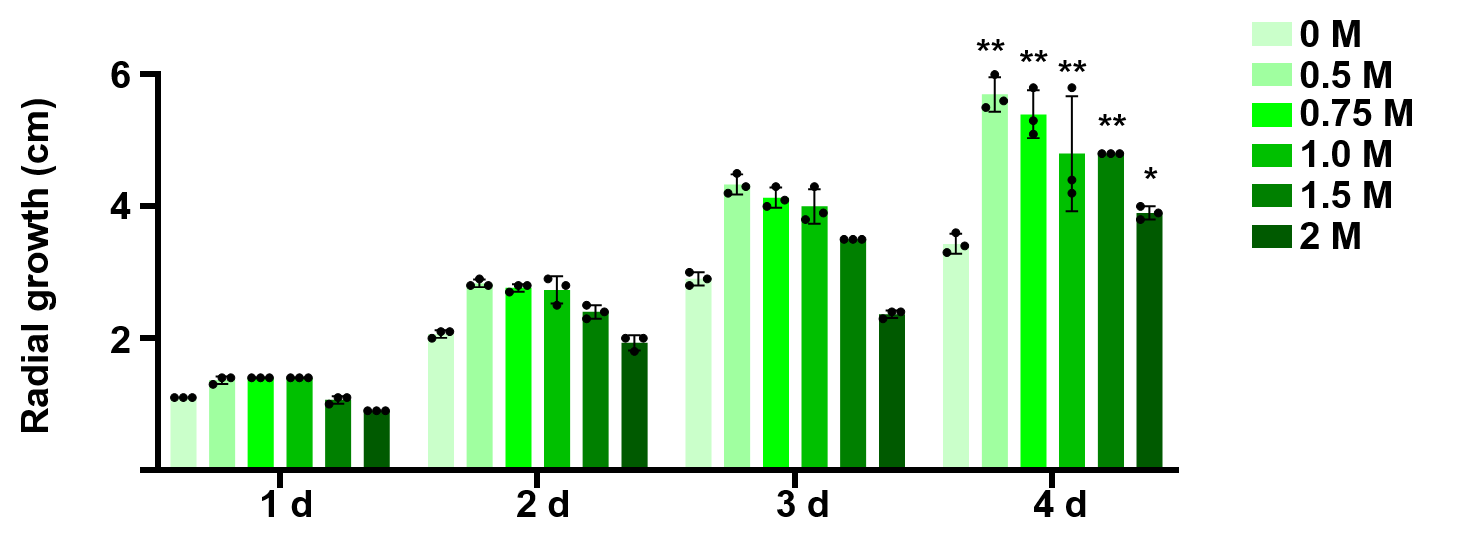

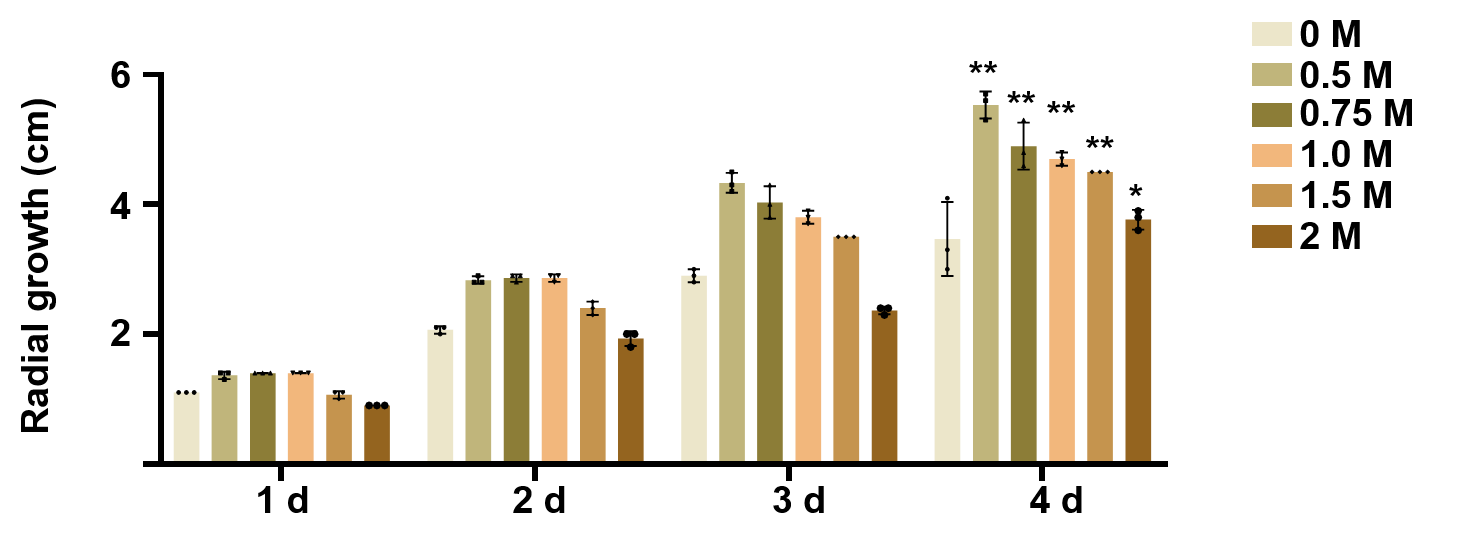


**c**

**b**

**Figure S2** Growth rate of *A. sydowii* from different sources on PDA within 7 days. **a,** *A. sydowii* DM1. **b,** *A. sydowii* SDM1. **c,** *A. sydowii* L-5. The data is the mean of three replicates. *A. sydowii* cultured under 0 M NaCl was used as control. Error bars in column graphs represent standard deviation of the mean. Asterisks represent statistically significant differences determined by one-way ANOVA (n.s., p>0.05, *, p<0.1, **, p<0.01, ***, p<0.001, ****, p<0.0001).

**Fig. S3**

**a**

**b**

**c**

**c**

**b**

**a**


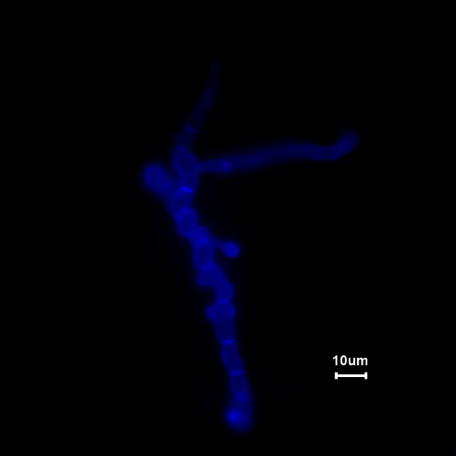

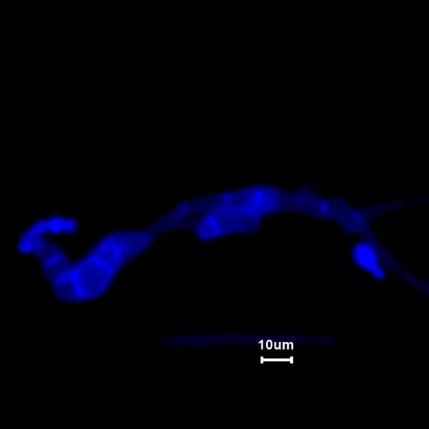


**Fig. S3** Hyphae of *A. sydowii* stained with fluorescent dye under elevated HP. a, *A. sydowii* SDM1 stained with CFW under 20 MPa. b, *A. sydowii* L-5 with CFW under 20 MPa. c, *A. sydowii* DM1 stained with CFW-PI under 40 MPa.

**Fig. S4**

**Fig. S4** Transcriptome overview. a, principal component analysis (PCA) of the gene expression profiles in different groups. Princomp function in RStudio 2022.12.0 performs PCA analysis and figure was were drawn using ggplot2 package. (b-c), numbers of differential expression genes (log_2_ foldchange > 1.5 or < -1.5) showed in venn diagram and bar plot. A total of 2410 DEGs were detected in *A. sydowii* DM1 cultured under 20 MPa, including 1366 up-regulated genes and 1044 down-regulated genes, while the magnitude doubled in the group of 40 MPa (5176 DEGs with 3271 up-regulated and 1905 down-regulated genes). Data was analyzed in Draw Venn Diagram (https://bioinformatics.psb.ugent.be/webtools/Venn/). M1 represents *A. sydowii* DM1 cultured under 0.1 MPa; M20 represents *A. sydowii* DM1 cultured under 20 MPa; M40 represents *A. sydowii* DM1 cultured under 40 MPa.


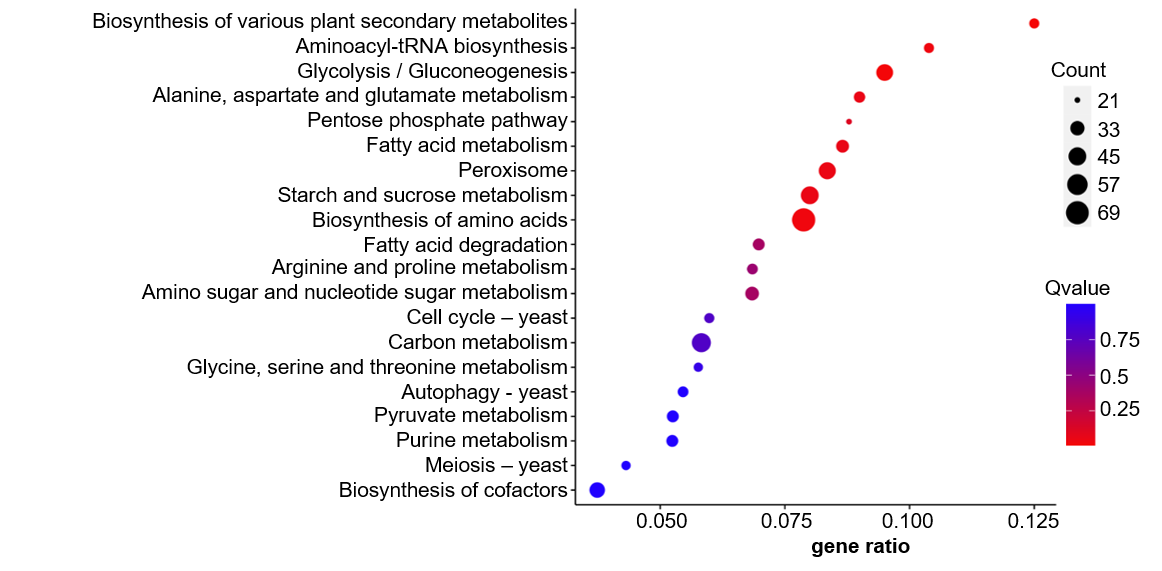
**Fig. S5**

**Fig. S5** Top 20 terms in KEGG enriched from commonly share genes (a total of 1530 genes) in two groups, which mainly enriched in biosynthesis of amino acids, carbohydrate metabolism (e.g., carbon metabolism, glycolysis, fatty acid metabolism) and cell process (e.g., cell cycle, autophagy, meiosis). Figure was drawn by ImageGP (https://www.bic.ac.cn/BIC/)

**Fig. S6**

**b**

**a**

**b**

**a**

**Fig. S6** Analysis of the DEGs of RNA-seq data of *A. sydowii* DM1 cultured under 20 MPa and 40 MPa, respectively. a, GO enrichment analysis on the set of DEGs under 20 MPa. b, GO enrichment analysis on the set of DEGs under 40 MPa. c, KEGG enrichment analysis on the set of DEGs under 20 MPa. d, KEGG enrichment analysis on the set of DEGs under 40 MPa. Enrichment terms analyzed in all detected genes from two groups were broadly in line with each other, mainly accounted by cellular anatomical entity, metabolic process, binding and catalytic activity in GO, carbohydrate metabolism, amino acid metabolism and transport and catabolism in KEGG. This graph was generated using GraphPad Prism 8.2.1.

**c**

**d**

**Fig. S7**

**Fig. S7** Expression levels of differently expressed genes validated by RT-qPCR. a, 7 genes were selected to verify the results of RNA-Seq by RT-qPCR. The selected genes belong to the core genes contributed to the proposal mechanisms in our study. Corroborating to this RNA-Seq analysis was our results of the similar tendency of DEGs during the post-pressurization time. (b-c), the value correlations between RNA-seq and RT-qPCR in 20 MPa (b) and 40 MPa group (c). R^2^ indicates the R-square of the regression line. The correlations between RNA-Seq and RT-qPCR exhibited well (R^2^ > 0.9). Error bars in column graphs represent standard deviation of the mean. The detailed analysis method is as described in Material and Method. Figure was plotted in GraphPad Prism 8.2.1.

**Fig. S8**

**Fig. S8** Fold change of marker genes in three *A. sydowii* under different HHP. The data is the mean of three replicates. *A.sydowii* DM1 was used as control to compare the statistical significance between the groups. Error bars in column graphs represent standard deviation of the mean. Asterisks represent statistically significant differences determined by one-way ANOVA (n.s., p>0.05, ***, p<0.001, ****, p<0.0001).

**Fig. S9**

**Fig. S9** Information of DEGs in carbohydrate metabolism. The number of up-regulated genes was significantly more than that of down-regulated genes (97+88 > 44+66); The top three items for up-regulated gene enrichment are listed: KO00520 (amino sugar and nucleotide sugar metabolism), KO00052 (galactose metabolism), KO00010 (glycolysis); The top three items for down-regulated gene enrichment are listed: KO00500 (starch and sucrose metabolism), KO00999 (biosynthesis of various plant secondary metabolites), KO00460 (cyanoamino acid metabolism)
