# Supplementary material for: Insight into the adaptation mechanisms of high hydrostatic pressure in physiology and metabolism of hadal fungi from the deepest ocean sediment
Source: mSystems. 2023 Dec 20;9(1):e01085-23. doi: 10.1128/msystems.01085-23 (PMC10804941; doi:10.1128/msystems.01085-23)
Supplement: Tables S1 and S2 — Primers and statistical summary of the results of RNA-seq data. [file msystems.01085-23-s0002.docx]

| **gene** | **Primer name** | **sequence (5'-3')** | **TM (°C)** | **Th (°C)** | **Conc. (μM)** | **Purpose** |
| --- | --- | --- | --- | --- | --- | --- |
| *ITS* | ITS1 | TCCGTAGGTGAACCTGCGG | 68 | 55 | 10 | phylogeny marker |
|  | ITS4 | TCCTCCGCTTATTGATATGC | 59 |  | 10 |  |
| *benA* | Bt2a | GGTAACCAAATCGGTGCTGCTTTC | 65 | 60 | 10 | phylogeny marker/reference gene |
|  | Bt2b | ACCCTCAGTGTAGTGACCCTTGGC | 70 |  | 10 |  |
| *cam* | CMD5 | CCGAGTACAAGGARGCCTTC | 63 | 56 | 10 | phylogeny marker |
|  | CMD6 | CCGATRGAGGTCATRACGTGG | 61 |  | 10 |  |
| *hog1* | hog1F | GGACGATGTGATCCAGACCAT | 64 | 58 | 10 | qPCR |
|  | hog1R | CTCTCGCTTCGGTAGTGACTT | 63 |  | 10 |  |
| *chs1* | CHS1F | ACGACTTCACACTGCGGAAT | 64 | 59 | 10 | qPCR |
|  | CHS1R | TTCCCGTCGATGTCGTGTTT | 64 |  | 10 |  |
| *fadD* | fadD1F | CCTCGGACATCGTCACCATCA | 66 | 60 | 10 | qPCR |
|  | fadD1R | CAGTCGGCTTGAGCAACTTGAG | 65 |  | 10 |  |
| *gas1* | gas1F | TGCGACGAACGACGATAAGAGT | 65 | 60 | 10 | qPCR |
|  | gas1R | CGACGACGGAGACAAGACCAT | 66 |  | 10 |  |
| *pgk1* | pgk1F | GCGACGTCATTTTCACGGAG | 64 | 59 | 10 | qPCR |
|  | pgk1R | AGCAACCTTCTCCTTGTCGG | 65 |  | 10 |  |
| *sho1* | sho1F | CGATGTCGACCGACGTGTAT | 64 | 59 | 10 | qPCR |
|  | sho1R | TCAGCATCCAGCAGTTCGTT | 64 |  | 10 |  |
| *hsl7* | hsl7F | TTGTGACATCCGCATGGAGC | 66 | 61 | 10 | qPCR |
|  | hsl7R | GAAGAAGACGTCCACGCCAG | 66 |  | 10 |  |

**Supplementary Table S1** Primers information used in this work.

Abbreviations: **Tm** – Calculated Melting Temperature of primer, **Th** – Actual Annealing Temperature used in PCR, **Conc**. – Primer concentration in the assay.

**Supplementary** **Table S2** Statistical summary of the results of RNA-seq data. (M1 represents *A. sydowii* DM1 cultured under 0.1 MPa; M20 represents *A. sydowii* DM1 cultured under 20 MPa; M40 represents *A. sydowii* DM1 cultured under 40 MPa)

| **Sample** **name** | **total raw** **read (M)** | **total clean read (M)** | **total clean base** | **clean read**  **q20 (%)** | **clean read**  **q30 (%)** | **clean read ratio**  **(%)** |
| --- | --- | --- | --- | --- | --- | --- |
| M1-1 | 43.69 | 42.04 | 6.31 | 96.33 | 89.9 | 96.21 |
| M1-2 | 43.69 | 42.02 | 6.3 | 96.42 | 90.11 | 96.18 |
| M1-3 | 43.69 | 41.94 | 6.29 | 96.56 | 90.48 | 96 |
| M20-1 | 43.69 | 42.07 | 6.31 | 96.03 | 89.13 | 96.3 |
| M20-2 | 45.44 | 43.31 | 6.5 | 96.44 | 89.96 | 95.31 |
| M20-3 | 45.44 | 42.91 | 6.44 | 96.55 | 90.28 | 94.45 |
| M40-1 | 45.44 | 43.47 | 6.52 | 97.43 | 92.19 | 95.66 |
| M40-2 | 45.44 | 43.19 | 6.48 | 96.5 | 90.07 | 95.05 |
| M40-3 | 45.44 | 42.91 | 6.44 | 96.57 | 90.33 | 94.43 |

An average total of 43.69, 45.44 and 45.44 million raw reads per library, respectively. The clean read ratio raged from 94-96%.
