# Supplementary material for: Insight into the adaptation mechanisms of high hydrostatic pressure in physiology and metabolism of hadal fungi from the deepest ocean sediment
Source: mSystems. 2023 Dec 20;9(1):e01085-23. doi: 10.1128/msystems.01085-23 (PMC10804941; doi:10.1128/msystems.01085-23)
Supplement: Table S3 — Transcriptomic expression of Aspergillus sydowii DM1's genes. [file msystems.01085-23-s0003.docx]

**Supplementary Table S3** Transcriptomic expression of *Aspergillus sydowii* DM1’s genes related to this work at 20 MPa and 40 MPa compared to 0.1 MPa.

| **process** | **gene ID** | **gene name** | **log_2_ FC in 20 MPa group** | **Q value in 20 MPa group** | **log_2_ FC in 40 MPa group** | | **Q value in 40 MPa group** | **description** |
| --- | --- | --- | --- | --- | --- | --- | --- | --- |
| cell wall component | Unigene3043_All | *csxA* | 0.896468067 | 0.000314017 | 1.523863711 | | 1.50E-13 | exo-1,4-beta-D-glucosaminidase |
|  | CL808.Contig2_All | *csn* | 2.19360632 | 1.36E-07 | 0.198630887 | | 0.783072066 | chitosanase |
|  | CL446.Contig4_All | *E3.2.1.14* | 11.74324116 | 2.62E-19 | 11.638647 | | 4.12E-16 | chitinase [EC:3.2.1.14] |
|  | CL768.Contig27_All |  | 10.89087694 | 4.50E-14 | 7.926516958 | | 5.16E-07 |  |
|  | CL768.Contig19_All |  | 10.87806634 | 3.12E-17 | 9.080195789 | | 7.30E-08 |  |
|  | CL65.Contig24_All |  | 9.474644034 | 2.13E-07 | 8.120325066 | | 0.059701484 |  |
|  | CL446.Contig11_All |  | 9.16973175 | 1.72E-06 | 4.432020364 | | 0.415984674 |  |
|  | CL768.Contig36_All |  | 8.364654851 | 5.58E-08 | 9.936729944 | | 4.75E-12 |  |
|  | CL768.Contig4_All |  | 8.341829308 | 4.74E-08 | 3.259024425 | | 0.209761569 |  |
|  | Unigene1664_All |  | 5.610732829 | 0.000274656 | 4.546029316 | | 0.006260728 |  |
|  | CL768.Contig31_All |  | 5.224502784 | 0.005947392 | 2.704058916 | | 0.561164963 |  |
|  | CL768.Contig1_All |  | 4.405738278 | 3.75E-23 | 0.672458474 | | 0.416959502 |  |
|  | Unigene1629_All |  | 4.326509078 | 3.61E-16 | 2.263947696 | | 0.002052907 |  |
| **process** | **gene ID** | **gene name** | **log_2_FC in 20 MPa group** | **Qvalue in 20 MPa group** | **log_2_FC in 40 MPa group** | | **Qvalue in 40 MPa group** | **description** |
| cell wall component | CL768.Contig6_All | *E3.2.1.14* | 3.664991957 | 5.01E-150 | 0.366214474 | | 0.183830312 | chitinase [EC:3.2.1.14] |
|  | Unigene1632_All |  | 2.174520833 | 1.70E-07 | 0.894007628 | | 0.128816828 |  |
|  | Unigene2539_All |  | 1.953219024 | 8.16E-05 | 2.475095481 | | 1.05E-07 |  |
|  | CL65.Contig12_All |  | 1.898191299 | 0.029690349 | 2.101807579 | | 0.01136582 |  |
|  | Unigene1633_All |  | 1.666257212 | 0.001049416 | 0.784585163 | | 0.141450984 |  |
|  | Unigene1650_All |  | 1.575280285 | 0.002715526 | 1.493041775 | | 0.003593859 |  |
|  | CL768.Contig14_All |  | -3.980836269 | 9.29E-10 | -1.961927129 | | 0.127050133 |  |
|  | CL446.Contig23_All |  | -10.63965682 | 5.67E-12 | -10.64389902 | | 7.50E-12 |  |
|  | CL1418.Contig4_All | *chs1* | -1.459570985 | 8.00E-37 | -1.774845445 | | 1.34E-14 | chitin synthase [EC:2.4.1.16] |
|  | CL1418.Contig2_All |  | -1.460204284 | 2.71E-05 | -1.642847378 | | 7.59E-07 |  |
|  | CL1418.Contig12_All |  | -1.834901691 | 1.94E-28 | -1.840461035 | | 2.11E-46 |  |
|  | CL1578.Contig3_All |  | -1.889624889 | 0.006986974 | -1.041900901 | | 0.271536392 |  |
|  | CL634.Contig1_All |  | -2.025206371 | 0.009471524 | -1.356786493 | | 0.546414849 |  |
|  | CL3519.Contig3_All |  | -2.216179322 | 0.007329419 | -1.169762691 | | 0.296933334 |  |
|  | CL634.Contig2_All |  | -4.506517796 | 5.28E-05 | -3.505047035 | | 0.174621284 |  |
| **process** | **gene ID** | **gene name** | **log_2_FC in 20 MPa group** | **Qvalue in 20 MPa group** | **log_2_FC in 40 MPa group** | | **Qvalue in 40 MPa group** | **description** |
| cell wall component | CL278.Contig43_All | *gas1* | 10.21790989 | 6.42E-15 | 10.05413647 | | 1.44E-11 | 1,3-beta-glucanosyltransferase |
|  | CL278.Contig18_All |  | 8.610238506 | 2.63E-10 | 10.5096901 | | 2.76E-13 |  |
|  | CL278.Contig29_All |  | 7.396701563 | 3.33E-05 | 6.701921677 | | 0.00056526 |  |
|  | CL2014.Contig4_All |  | 7.329855532 | 2.81E-06 | 8.602331118 | | 3.64E-06 |  |
|  | CL278.Contig48_All |  | -1.787360562 | 4.22E-09 | -1.720823224 | | 7.23E-09 |  |
|  | CL278.Contig30_All |  | -3.899547022 | 0.00060669 | -2.889895657 | | 0.234926489 |  |
|  | CL717.Contig12_All | *PIG-W* | 4.368343 | 0.039930266 | 5.814888764 | | 0.093046812 | glucosaminylphosphatidylinositol acyltransferase |
|  | CL717.Contig8_All |  | 5.281414 | 0.002774545 | 6.861343458 | | 1.77E-06 |  |
|  | CL717.Contig9_All |  | 10.27032 | 4.32E-15 | 10.61067556 | | 5.95E-13 |  |
|  | CL474.Contig2_All | *PIG-X* | 1.654281697 | 7.81E-05 | 2.526189015 | | 4.67E-19 | GPI mannosyltransferase 1 subunit X |
|  | CL3.Contig2_All | *PIG-V* | 1.669643602 | 0.000195245 | 1.403186325 | | 0.003194174 | GPI mannosyltransferase 2 [EC:2.4.1.-] |
|  | CL295.Contig8_All | *PIG-B* | -10.29995561 | 1.15E-13 | -10.30409065 | | 1.36E-13 | GPI mannosyltransferase 3 [EC:2.4.1.-] |
|  | CL677.Contig33_All | *MAN2C1* | 9.631215762 | 1.70E-08 | 5.734650906 | | 0.265751462 | alpha-mannosidase |
|  | Unigene5437_All | *MANBA, manB* | 4.962609594 | 1.97E-15 | 6.084930867 | | 1.36E-29 | beta-mannosidase |
| **process** | **gene ID** | **gene name** | **log_2_FC in 20 MPa group** | **Qvalue in 20 MPa group** | **log_2_FC in 40 MPa group** | **Qvalue in 40 MPa group** | | **description** |
| cell wall component | CL1657.Contig2_All | *och1* | -2.932589168 | 3.33E-26 | -2.398988055 | | 1.43E-27 | alpha 1,6-mannosyltransferase |
|  | CL597.Contig19_All | *pmt* | -1.760238349 | 0 | -2.58589335 | | 4.19E-276 | dolichyl-phosphate-mannose-protein mannosyltransferase |
|  | CL510.Contig21_All | *alg1* | 7.976735971 | 0.000713297 | 3.694969931 | | 0.51534597 | beta-1,4-mannosyltransferase |
|  | Unigene4092_All | *alg3* | 1.536758444 | 5.48E-10 | 1.501747313 | | 1.08E-11 | alpha-1,3-mannosyltransferase |
|  | Unigene1272_All | *alg7* | 1.560906762 | 0.000240465 | 0.702522684 | | 0.208615775 | UDP-N-acetylglucosamine--dolichyl-phosphate N-acetylglucosaminephosphotransferase |
|  | CL1612.Contig1_All | *ktr* | 2.344570577 | 3.24E-05 | 1.795913562 | | 0.003090297 | mannosyltransferase |
|  | CL1590.Contig3_All | *chnB* | -1.537257909 | 0.00049674 | -2.490786636 | | 0.414523832 | cyclohexanone monooxygenase |
|  | Unigene3567_All | *-* | -3.794984543 | 0.004822623 | -3.66628308 | | 0.006874241 | fungal hydrophobin |
| septum formation | CL644.Contig8_All | *myo1* | -2.020256875 | 0.003679333 | 0.205384625 | | 0.596447596 | myosin I; myosin complex |
|  | CL644.Contig10_All |  | -6.123266729 | 0.000955371 | -0.735672345 | | 0.798868133 |  |
|  | CL68.Contig5_All | *CAPZA* | -0.51925807 | 0.836350919 | 2.925561548 | | 3.09E-05 | capping protein (actin filament) muscle Z-line, alpha |
|  | CL954.Contig9_All | *rho* | -1.605989689 | 0.044201863 | -0.321706442 | | 0.746042792 | GTP-binding protein rho3 |
|  | CL779.Contig5_All | *-* | -1.45557484 | 0.005212423 | -11.26753502 | | 9.07E-16 | bud site selection protein BUD4 |
| **process** | **gene ID** | **gene name** | **log_2_FC in 20 MPa group** | **Qvalue in 20 MPa group** | **log_2_FC in 40 MPa group** | | **Qvalue in 40 MPa group** | **description** |
| septum formation | CL779.Contig1_All | *-* | -0.603827366 | 0.061445657 | -2.464617547 | | 8.11E-16 | bud site selection protein BUD4 |
| MAPK pathway | CL384.Contig7_All | *wcs1-3* | -2.210804129 | 0.000546674 | -2.474982354 | | 0.205343029 | cell wall integrity and stress response component |
|  | CL1082.Contig3_All | *sac7* | 2.52 | 0.002129244 | 0.4719 | | 0.706313411 | GTPase-activating protein |
|  | CL3012.Contig7_All | *mih1* | -9.75 | 0.012923924 | -1.387200619 | | 0.812187159 | M-phase inducer tyrosine phosphatase |
|  | CL370.Contig5_All | *erk* | -9.744256895 | 9.76E-13 | -1.881782787 | | 0.377097209 | mitogen-activated protein kinase 1/3 |
|  | CL2762.Contig2_All | *pkc1* | -1.615415355 | 0.000418135 | -1.939138666 | | 2.74E-05 | classical protein kinase C alpha type |
|  | CL2762.Contig3_All |  | -1.95397116 | 7.20E-15 | -2.200300861 | | 9.55E-05 |  |
|  | CL2368.Contig2_All | *sln1* | 1.649192867 | 2.47E-10 | 2.563550133 | | 4.97E-24 | osomolarity two-component system, sensor histidine kinase |
|  | CL5.Contig2_All |  | 3.764866456 | 3.31E-50 | 7.233437376 | | 0 |  |
|  | CL5.Contig3_All |  | 4.279698336 | 0.000173716 | 6.989578034 | | 3.66E-27 |  |
|  | CL1573.Contig5_All | *sho1* | 1.72 | 1.98E-09 | 3.110667098 | | 8.97E-54 | osmosensor |
|  | CL392.Contig4_All | *ste20* | 4.78 | 0.034790763 | 4.887139468 | | 0.026273002 | p21-activated kinase 1 |
|  | Unigene6947_All | *hog1* | -2.246659873 | 0.000339313 | -7.526775466 | | 2.85E-08 | p38 MAP kinase |
|  | CL438.Contig12_All | *ypd1* | -2.797852604 | 4.35E-14 | -1.874375112 | | 1.07E-10 | osomolarity two-component system |
| **process** | **gene ID** | **gene name** | **log_2_FC in 20 MPa group** | **Qvalue in 20 MPa group** | **log_2_FC in 40 MPa group** | | **Qvalue in 40 MPa group** | **description** |
| MAPK pathway | CL438.Contig8_All | *ypd1* | 8.425830368 | 0.044333399 | 8.665056212 | | 1.39E-08 | osomolarity two-component system |
| cell cycle | CL1069.Contig1_All | *hsl7* | -9.11948774 | 5.86E-06 | -9.134253243 | | 6.53E-06 | type II protein arginine methyltransferase |
|  | CL1213.Contig13_All | *scf* | -1.583063799 | 4.33E-06 | -2.300727938 | | 0.096844198 | cullin 1 |
|  | Unigene5615_All | *cdc55* | 7.244161448 | 0.00857647 | 7.053632786 | | 0.00037953 | serine/threonine-protein phosphatase 2A regulatory subunit B |
|  | CL2697.Contig1_All | *dum1* | 1.706631145 | 0.000301098 | 1.806298678 | | 6.11E-05 | serine/threonine-protein kinase CHEK2 |
|  | CL3263.Contig2_All | *bub1* | 6.033389163 | 0.003411086 | 7.192800905 | | 1.89E-05 | checkpoint serine/threonine-protein kinase |
|  | CL1045.Contig8_All | *orc* | 1.719454455 | 0.004465104 | 3.60921027 | | 2.09E-15 | origin recognition complex subunit 2 |
|  | Unigene1957_All | *tem1* | -1.853638104 | 6.86E-88 | -2.346574044 | | 4.15E-97 | gtp-binding protein of the ras superfamily involved in termination of M-phase |
|  | CL1973.Contig3_All | *rad24* | -1.167204322 | 0.043530029 | -2.621892828 | | 0.000341844 | cell cycle checkpoint protein |
|  | CL1973.Contig4_All |  | -1.278206298 | 0.000167856 | -2.390095973 | | 0.002792075 |  |
| TCA cycle | Unigene4105_All | *gltA* | 2.28141564 | 1.21E-79 | 3.051976983 | | 4.19E-87 | citrate synthase |
|  | CL3143.Contig4_All |  | 1.59809557 | 5.96E-15 | 0.900648057 | | 0.077577033 |  |
|  | CL2385.Contig2_All | *sdh4* | 8.833385 | 1.29E-35 | 9.797961 | | 5.67E-45 | succinate dehydrogenase |
| **process** | **gene ID** | **gene name** | **log_2_FC in 20 MPa group** | **Qvalue in 20 MPa group** | **log_2_FC in 40 MPa group** | | **Qvalue in 40 MPa group** | **description** |
| TCA cycle | Unigene3854_All | *sdh4* | 5.228313 | 0.004711034 | 6.310307 | | 0.000112641 | succinate dehydrogenase |
|  | CL2205.Contig3_All |  | 2.502172 | 0.0084985 | 3.640308 | | 3.72E-06 |  |
|  | CL2205.Contig1_All |  | 2.016354 | 9.93E-09 | 1.662597 | | 2.71E-05 |  |
|  | CL2205.Contig6_All |  | 1.624557 | 0.027306957 | -0.06571 | | 0.96119976 |  |
|  | CL765.Contig1_All |  | -4.12766 | 0.024165205 | 0.628185 | | 0.708629978 |  |
|  | CL2241.Contig4_All | *mdh2* | 1.989394823 | 1.39E-08 | 2.12858747 | | 7.54E-14 | malate dehydrogenase |
| oxidative phosphorylation | CL1103.Contig1_All | *ppa* | -2.01285 | 3.12E-07 | -3.5113 | | 4.32E-08 | inorganic pyrophosphatase |
|  | CL1103.Contig5_All |  | -2.39318 | 0.00136086 | -1.96325 | | 0.008243494 |  |
|  | CL1103.Contig6_All |  | -2.20791 | 0.010637797 | -3.00583 | | 0.013082493 |  |
|  | CL1549.Contig7_All | *atp2* | -2.398371033 | 4.21E-06 | -1.549481381 | | 3.56E-07 | F-type H+-transporting ATPase subunit beta |
|  | CL1331.Contig1_All | *atp6N* | -1.629088349 | 5.65E-15 | -1.280801863 | | 2.39E-08 | V-type H+-transporting ATPase subunit a |
|  | CL2638.Contig1_All | *ptm* | -2.135347667 | 5.07E-34 | -2.581291221 | | 6.88E-12 | H+-transporting ATPase |
|  | CL2638.Contig2_All |  | -3.116448994 | 2.20E-20 | -3.828464313 | | 3.93E-30 |  |
|  | CL2638.Contig3_All |  | -1.288294591 | 0 | -1.807727968 | | 5.95E-227 |  |
| **process** | **gene ID** | **gene name** | **log_2_FC in 20 MPa group** | **Qvalue in 20 MPa group** | **log_2_FC in 40 MPa group** | | **Qvalue in 40 MPa group** | **description** |
| oxidative phosphorylation | CL2638.Contig4_All | *ptm* | -1.688315929 | 2.88E-10 | -1.722061064 | | 8.33E-18 | H+-transporting ATPase |
|  | CL1148.Contig7_All | *cox6A* | 10.62510387 | 4.40E-16 | 10.99273824 | | 2.41E-05 | cytochrome c oxidase subunit 6a |
|  | CL209.Contig3_All | *cox5B* | 2.475819709 | 0.000242205 | 1.727583278 | | 7.56E-05 | cytochrome c oxidase subunit 5b |
|  | CL1002.Contig2_All | *petC* | 1.757453469 | 4.91E-13 | 2.724327227 | | 3.94E-24 | ubiquinol-cytochrome c reductase cytochrome c1 subunit |
|  | CL1002.Contig6_All |  | 11.02584475 | 9.37E-16 | 13.02775645 | | 8.98E-26 |  |
| glycolysis | CL1730.Contig4_All | *pdhC* | -2.360472742 | 1.38E-14 | -1.971552405 | | 1.02E-19 | pyruvate dehydrogenase |
|  | CL1730.Contig1_All |  | -2.172265218 | 5.24E-26 | -2.329000974 | | 9.49E-24 |  |
|  | CL519.Contig5_All | *fumC* | -2.928816856 | 1.81E-06 | -4.274331365 | | 0.006588022 | fumarate hydratase |
|  | CL63.Contig12_All | *pyk* | 7.686519411 | 1.03E-06 | 5.528157592 | | 0.28712474 | pyruvate kinase |
|  | CL63.Contig18_All |  | 2.60416607 | 1.03E-06 | 2.539387694 | | 0.00052676 |  |
|  | CL63.Contig1_All |  | 3.499436912 | 2.60E-05 | 3.167911129 | | 0.000187224 |  |
|  | CL63.Contig27_All |  | 1.743821455 | 1.53E-08 | 1.602794918 | | 4.32E-08 |  |
|  | CL3696.Contig1_All | *eno* | 4.394361299 | 0.047892872 | 0.314940785 | | 0.959202393 | enolase |
|  | CL221.Contig10_All | *pgk* | 10.75298588 | 2.30E-12 | 12.19618946 | | 4.49E-23 | phosphoglycerate kinase |
| **process** | **gene ID** | **gene name** | **log_2_FC in 20 MPa group** | **Qvalue in 20 MPa group** | **log_2_FC in 40 MPa group** | | **Qvalue in 40 MPa group** | **description** |
| glycolysis | Unigene4596_All | *pgm* | -2.10667 | 8.72E-15 | -1.8996 | | 2.99E-11 | phosphoglucomutase |
|  | CL698.Contig6_All |  | -2.1044 | 0.035845243 | -1.4927 | | 0.008283542 |  |
|  | CL2009.Contig10_All |  | 1.583356 | 0.041242987 | -0.23445 | | 0.93245903 |  |
|  | CL2523.Contig3_All |  | 1.761404 | 0.000187739 | -1.43399 | | 0.49859676 |  |
|  | CL2009.Contig8_All |  | 1.798011 | 1.05E-07 | -0.51271 | | 0.367551404 |  |
|  | Unigene2965_All |  | 2.280773 | 0.003485859 | 2.569673 | | 0.000198561 |  |
|  | CL3265.Contig3_All |  | 2.341855 | 3.63E-17 | 4.008264 | | 1.05E-72 |  |
|  | CL2009.Contig2_All |  | 2.469359 | 1.76E-11 | 0.289392 | | 0.68225747 |  |
|  | CL1652.Contig11_All |  | 2.685129 | 3.01E-08 | -7.49913 | | 1.30E-05 |  |
|  | CL3018.Contig2_All |  | 2.709266 | 7.18E-06 | 3.139938 | | 1.18E-07 |  |
|  | CL2523.Contig1_All |  | 2.733828 | 0.006197709 | 0.231464 | | 0.934921875 |  |
|  | CL2161.Contig4_All |  | 2.892852 | 6.06E-05 | 0.446156 | | 0.779243924 |  |
|  | CL3018.Contig3_All |  | 3.15893 | 7.66E-23 | 3.012464 | | 2.98E-16 |  |
|  | Unigene4760_All |  | 3.436571 | 1.24E-06 | 4.663294 | | 1.62E-17 |  |
| **process** | **gene ID** | **gene name** | **log_2_FC in 20 MPa group** | **Qvalue in 20 MPa group** | **log_2_FC in 40 MPa group** | | **Qvalue in 40 MPa group** | **description** |
| glycolysis | CL2161.Contig5_All | *pgm* | 3.933681 | 1.23E-20 | 2.971756 | | 4.27E-08 | phosphoglucomutase |
|  | Unigene3213_All |  | 5.729653 | 5.36E-13 | 0.340087 | | 0.873646448 |  |
|  | CL2484.Contig4_All |  | 8.021655 | 1.94E-07 | 5.561058 | | 0.01361641 |  |
|  | CL2256.Contig1_All |  | 8.725266 | 2.07E-10 | 7.471729 | | 3.47E-07 |  |
|  | CL2523.Contig2_All |  | 9.177536 | 3.75E-09 | 7.34368 | | 0.002800684 |  |
|  | CL1652.Contig12_All |  | 9.704416 | 6.01E-14 | - | | - |  |
|  | CL423.Contig1_All | *pfk* | -9.828948268 | 0.009392694 | -9.806476685 | | 0.0098859 | 6-phosphofructokinase 1 |
|  | CL423.Contig4_All |  | -1.645274439 | 2.93E-13 | -1.807222847 | | 2.02E-58 |  |
|  | CL1993.Contig1_All | *E5.1.3.15* | 7.62224118 | 0.005321137 | 7.730746563 | | 0.002863894 | glucose-6-phosphate 1-epimerase |
|  | CL1993.Contig2_All |  | 8.972182403 | 1.35E-08 | 12.70383865 | | 8.88E-24 |  |
|  | CL1993.Contig3_All |  | 1.876554333 | 1.81E-13 | 1.420936217 | | 2.05E-20 |  |
|  | CL953.Contig1_All |  | 1.64742614 | 0.002299315 | 1.308679325 | | 0.023133976 |  |
|  | CL1733.Contig1_All | *GAPDH,* | -1.719651765 | 0.011884495 | -1.10108442 | | 0.152481607 | glyceraldehyde 3-phosphate dehydrogenase |
|  | CL1733.Contig8_All |  | -2.009253437 | 4.06E-05 | -6.086184453 | | 5.74E-11 |  |
| **process** | **gene ID** | **gene name** | **log_2_FC in 20 MPa group** | **Qvalue in 20 MPa group** | **log_2_FC in 40 MPa group** | | **Qvalue in 40 MPa group** | **description** |
| glycolysis | Unigene837_All | *pdhD* | 2.476708506 | 1.31E-16 | 3.907321235 | | 3.93E-40 | dihydrolipoamide dehydrogenase |
| fatty acid metabolism | CL1610.Contig5_All | *acaca* | 1.352462568 | 2.82E-05 | -0.342218381 | | 0.907764304 | acetyl-CoA carboxylase |
|  | CL71.Contig55_All | *acfs3* | 9.191446525 | 0.018849943 | - | | - | malonyl-CoA/methylmalonyl-CoA synthetase |
|  | CL71.Contig72_All |  | 9.076515218 | 0.001197465 | 8.670148473 | | 0.044484723 |  |
|  | CL71.Contig77_All |  | 4.843292193 | 0.01938511 | 6.307841187 | | 0.000166443 |  |
|  | CL1391.Contig3_All | *fadD* | 2.342552672 | 1.18E-22 | 4.146686252 | | 1.05E-57 | long-chain acyl-CoA synthetase |
|  | CL39.Contig9_All |  | 5.441504597 | 0.002848822 | 5.763512069 | | 0.000542197 |  |
|  | CL629.Contig21_All |  | 1.700415351 | 2.40E-43 | -1.116624588 | | 2.85E-15 |  |
|  | Unigene2496_All |  | 3.441650599 | 1.39E-07 | 4.46149404 | | 1.09E-12 |  |
|  | Unigene3893_All |  | 10.17103175 | 0.005264987 | 12.9210626 | | 1.82E-25 |  |
|  | Unigene4422_All |  | 2.254907282 | 0.027118784 | 2.306727491 | | 0.033731223 |  |
|  | Unigene4433_All |  | 3.194105695 | 0.000119242 | 4.809150275 | | 1.04E-10 |  |
|  | CL2701.Contig1_All | *acox* | 3.226310374 | 8.18E-89 | 4.01081078 | | 6.10E-81 | acyl-CoA oxidase |
|  | CL2701.Contig2_All |  | 2.2804254 | 1.75E-17 | 3.720893495 | | 7.28E-52 |  |
| **process** | **gene ID** | **gene name** | **log_2_FC in 20 MPa group** | **Qvalue in 20 MPa group** | **log_2_FC in 40 MPa group** | | **Qvalue in 40 MPa group** | **description** |
| fatty acid metabolism | CL352.Contig1_All | *acox* | 4.999802214 | 0.002111198 | 5.297374536 | | 0.002028467 | acyl-CoA oxidase |
|  | CL352.Contig4_All |  | 3.512097076 | 0.000223296 | 3.100103505 | | 0.033023568 |  |
|  | CL352.Contig6_All |  | 2.577455249 | 0.001094955 | 2.304668895 | | 0.005924753 |  |
|  | Unigene2582_All | *echs1* | 2.375174056 | 2.97E-08 | 2.277614802 | | 1.01E-05 | enoyl-CoA hydratase |
|  | CL2475.Contig1_All | *hadh* | -1.501580106 | 0.000894627 | -2.110987578 | | 2.52E-07 | 3-hydroxyacyl-CoA dehydrogenase |
|  | CL33.Contig1_All | *acadsb* | 2.360225354 | 6.29E-10 | 3.970027954 | | 9.15E-31 | short-chain 2-methylacyl-CoA dehydrogenase |
|  | CL33.Contig4_All |  | 2.283917817 | 2.00E-33 | 2.626571476 | | 1.00E-34 |  |
|  | CL1755.Contig4_All | *fabG* | 10.15210269 | 2.75E-13 | 10.70960766 | | 3.07E-16 | 3-oxoacyl-[acyl-carrier protein] reductase |
|  | CL238.Contig2_All |  | 5.246271946 | 1.00E-186 | 3.912149916 | | 7.91E-98 |  |
|  | CL3425.Contig2_All |  | 2.047460585 | 8.06E-09 | 1.81745808 | | 1.36E-06 |  |
|  | Unigene388_All |  | 1.774899502 | 0.040052364 | 4.507790012 | | 2.01E-13 |  |
|  | CL1755.Contig2_All |  | -1.835412201 | 2.78E-08 | -3.799400562 | | 7.33E-40 |  |
| glycerolipid metabolism | CL3449.Contig2_All | *gcy1* | 1.677672513 | 9.74E-10 | 2.591227791 | | 1.72E-22 | glycerol 2-dehydrogenase |
|  | CL1205.Contig1_All |  | 6.400404354 | 0.00119539 | - | | - |  |
| **process** | **gene ID** | **gene name** | **log_2_FC in 20 MPa group** | **Qvalue in 20 MPa group** | **log_2_FC in 40 MPa group** | | **Qvalue in 40 MPa group** | **description** |
| glycerolipid metabolism | CL2161.Contig5_All | *gcy1* | 3.933681345 | 1.23E-20 | 2.97175639 | | 4.27E-08 | glycerol 2-dehydrogenase |
|  | Unigene5836_All |  | 2.837577114 | 7.45E-19 | -0.590312371 | | 0.311777603 |  |
|  | CL1205.Contig4_All |  | 2.003169508 | 2.30E-188 | 0.235943121 | | 0.292581084 |  |
|  | CL710.Contig13_All | *acaa1* | 1.00082 | 2.46E-10 | 0.328737 | | 0.480988937 | acetyl-CoA acyltransferase 1 |
|  | CL710.Contig17_All |  | 2.302562 | 9.92E-07 | 1.655691 | | 0.008790348 |  |
|  | CL710.Contig18_All |  | -8.45908 | 8.82E-09 | 4.437989 | | 1.98E-65 |  |
|  | CL710.Contig8_All |  | 1.720387 | 0.00547424 | 1.417552 | | 0.032027236 |  |
|  | CL908.Contig1_All |  | -0.71415 | 0.001084934 | 0.75471 | | 9.34E-08 |  |
|  | Unigene3514_All |  | 1.013573 | 0.012437176 | 1.196774 | | 0.000118684 |  |
|  | Unigene3999_All |  | 1.200181 | 0.00018337 | 1.898917 | | 5.57E-09 |  |
|  | CL3290.Contig1_All | *aldh* | 4.276188289 | 4.89E-11 | -4.555014628 | | 0.915536197 | aldehyde dehydrogenase (NAD+) |
|  | CL1618.Contig1_All | *gpp* | 1.784505064 | 0.000270836 | -0.187966012 | | 0.9472975 | glycerol-1-phosphatase |
| alanine, aspartate and glutamate metabolism | CL1903.Contig8_All | *argG* | *3.548480563* | 0.000239282 | 5.196371664 | | 8.43E-11 | argininosuccinate synthase |
|  | CL2694.Contig3_All | *purA* | *1.965953234* | 4.28E-35 | 2.778752835 | | 1.45E-62 | adenylosuccinate synthase |
| **process** | **gene ID** | **gene name** | **log_2_FC in 20 MPa group** | **Qvalue in 20 MPa group** | **log_2_FC in 40 MPa group** | | **Qvalue in 40 MPa group** | **description** |
| alanine, aspartate and glutamate metabolism | CL2694.Contig6_All | *purA* | *1.578312398* | 1.24E-32 | 2.511910084 | | 9.10E-69 | adenylosuccinate synthase |
|  | Unigene5102_All |  | *1.426798009* | 2.83E-05 | 4.387861495 | | 4.92E-60 |  |
|  | CL419.Contig14_All | *gdh2* | *4.452521717* | 7.65E-11 | 4.508457129 | | 1.08E-08 | glutamate dehydrogenase |
|  | CL419.Contig5_All |  | *3.734232814* | 1.09E-07 | 4.962364764 | | 1.62E-15 |  |
|  | CL3439.Contig1_All | *gad1* | *1.217805511* | 4.05E-11 | 1.868390501 | | 1.10E-40 | glutamate decarboxylase |
|  | CL2523.Contig2_All | *abat* | *9.177536* | 3.75E-09 | 7.34368 | | 0.002800684 | 4-aminobutyrate aminotransferase |
|  | CL2523.Contig3_All |  | *1.761404* | 0.000187739 | -1.43399 | | 0.49859676 |  |
|  | CL2530.Contig1_All |  | *9.194353* | 2.27E-25 | 8.868072 | | 8.45E-22 |  |
|  | CL3018.Contig2_All |  | *2.709266* | 7.18E-06 | 3.139938 | | 1.18E-07 |  |
|  | CL3018.Contig3_All |  | *3.15893* | 7.66E-23 | 3.012464 | | 2.98E-16 |  |
|  | CL3265.Contig3_All |  | *2.341855* | 3.63E-17 | 4.008264 | | 1.05E-72 |  |
|  | CL426.Contig8_All |  | *1.278122* | 0.000919537 | 1.951515 | | 1.34E-06 |  |
|  | Unigene2400_All |  | *1.891575* | 0.000174212 | 2.07647 | | 0.000267628 |  |
|  | Unigene3213_All |  | *5.729653* | 5.36E-13 | 0.340087 | | 0.873646448 |  |
| **process** | **gene ID** | **gene name** | **log_2_FC in 20 MPa group** | **Qvalue in 20 MPa group** | **log_2_FC in 40 MPa group** | | **Qvalue in 40 MPa group** | **description** |
|  | Unigene4596_All | *abat* | *-2.10667* | 8.72E-15 | -1.8996 | | 2.99E-11 | 4-aminobutyrate aminotransferase |
| D-amino acids | CL592.Contig16_All | *aao* | *1.91547206* | 0.000211413 | 4.629809124 | | 6.24E-46 | D-amino-acid oxidase |
| response to oxdative stress | CL622.Contig1_All | *sod2* | 4.34855718 | 4.97E-76 | 5.019751121 | | 2.46E-38 | superoxide dismutase, Fe-Mn family |
|  | Unigene143_All | *CAT* | 2.265477143 | 0.004767855 | 5.2589346 | | 3.05E-17 | catalase |
|  | CL349.Contig14_All | *skn7* | 9.605620209 | 1.08E-13 | 9.016535766 | | 2.82E-09 | osomolarity two-component system, response regulator SKN7 |
|  | CL349.Contig40_All |  | 8.851641313 | 4.20E-14 | 8.491570904 | | 5.75E-11 |  |
|  | CL349.Contig41_All |  | 3.006297059 | 0.003356663 | 3.030455911 | | 0.009048784 |  |
|  | CL349.Contig7_All |  | 2.874216398 | 5.29E-41 | 2.27328976 | | 3.09E-22 |  |
|  | CL1589.Contig2_All | *E1.11.1.5* | 2.295861612 | 2.05E-25 | 4.499179194 | | 1.22E-81 | cytochrome c peroxidase |
|  | CL173.Contig4_All | *ahp1* | -3.400752186 | 4.52E-08 | -3.524633886 | | 0.000400675 | alkyl hydroperoxide reductase 1 |
| DNA recombination | CL1759.Contig4_All | *ku70* | 1.301956664 | 0.010696368 | 2.717676233 | | 1.46E-05 | ATP-dependent DNA helicase 2 subunit 1 |
|  | CL1759.Contig3_All |  | 2.364575176 | 4.27E-27 | 3.411930616 | | 4.94E-69 |  |
|  | CL1365.Contig13_All | *NSE4* | -1.844713347 | 0.007245899 | -1.73581339 | | 9.71E-08 | non-structural maintenance of chromosomes element 4 |
| heat shock protein | CL39.Contig7_All | *dnaK* | 11.09821009 | 2.29E-16 | 12.40837955 | | 7.99E-23 | molecular chaperone DnaK |
| **process** | **gene ID** | **gene name** | **log_2_FC in 20 MPa group** | **Qvalue in 20 MPa group** | **log_2_FC in 40 MPa group** | | **Qvalue in 40 MPa group** | **description** |
| heat shock protein | CL1344.Contig11_All | *HSPA1s* | 9.853924282 | 0.014985606 | 14.96730057 | | 4.06E-31 | heat shock 70kDa protein |
|  | CL1344.Contig2_All |  | -2.169141908 | 8.48E-37 | -2.48700711 | | 1.57E-48 |  |
| DNA repair | CL218.Contig3_All | *XPF* | -1.663048338 | 0.049044849 | -10.84484764 | | 1.32E-16 | DNA excision repair protein ERCC-4 |
|  | CL218.Contig20_All |  | 7.129932043 | 6.69E-05 | 7.18935969 | | 1.58E-05 |  |
|  | CL97.Contig1_All | *dnl4* | 9.701780411 | 9.24E-08 | 10.41411322 | | 0.004010711 | DNA ligase 4 |
|  | CL1759.Contig2_All | *ku70* | 1.481944 | 5.01E-07 | 1.339063 | | 1.50E-05 | ATP-dependent DNA helicase 2 subunit 1 |
|  | CL1759.Contig3_All |  | 2.364575 | 4.27E-27 | 3.411931 | | 4.94E-69 |  |
|  | CL1759.Contig4_All |  | 1.301957 | 0.010696368 | 2.717676 | | 1.46E-05 |  |
|  | CL710.Contig15_All | *rad57* | 8.513316004 | 9.74E-10 | - | | - | DNA repair protein |
|  | CL1707.Contig2_All | *EME1, MMS4* | 1.369177 | 1.50E-14 | 1.643883 | | 2.43E-16 | crossover junction endonuclease EME1 |
|  | Unigene943_All | *PIF1* | -1.741086869 | 0.002695917 | -4.58192557 | | 0.022279205 | ATP-dependent DNA helicase PIF1 |
