# Supplementary material for: Insight into the adaptation mechanisms of high hydrostatic pressure in physiology and metabolism of hadal fungi from the deepest ocean sediment
Source: mSystems. 2023 Dec 20;9(1):e01085-23. doi: 10.1128/msystems.01085-23 (PMC10804941; doi:10.1128/msystems.01085-23)
Supplement: Table S4 — Detailed information of top 10 terms in DEGs from all groups. [file msystems.01085-23-s0004.docx]

**Supplementary Table S4** Detailed information of top 10 terms in DEGs from all groups

| **Group** | **Pathway ID** | **Pathway Name** | **Level 1** | **Level 2** | **TCGN** | **TGN** | **Ratio** | **P value** | **Q value** |
| --- | --- | --- | --- | --- | --- | --- | --- | --- | --- |
| 20MPa | ko01230 | biosynthesis of amino acids | metabolism | global and overview maps | 90 | 876 | 0.1027 | 0.0267 | 0.1518 |
| 20MPa | ko00500 | starch and sucrose metabolism | metabolism | carbohydrate metabolism | 71 | 550 | 0.1291 | 0.0002 | 0.0032 |
| 20MPa | ko01200 | carbon metabolism | metabolism | global and overview maps | 70 | 841 | 0.0832 | 0.5662 | 0.9989 |
| 20MPa | ko00520 | amino sugar and nucleotide sugar metabolism | metabolism | carbohydrate metabolism | 67 | 453 | 0.1479 | 0.0000 | 0.0002 |
| 20MPa | ko01240 | biosynthesis of cofactors | metabolism | global and overview maps | 67 | 963 | 0.0696 | 0.9647 | 0.9989 |
| 20MPa | ko00010 | glycolysis | metabolism | carbohydrate metabolism | 59 | 421 | 0.1401 | 0.0001 | 0.0014 |
| 20MPa | ko00230 | purine metabolism | metabolism | nucleotide metabolism | 53 | 515 | 0.1029 | 0.0738 | 0.3190 |
| 20MPa | ko04146 | peroxisome | cellular processes | transport and catabolism | 52 | 491 | 0.1059 | 0.0506 | 0.2502 |
| 20MPa | ko00030 | pentose phosphate pathway | metabolism | carbohydrate metabolism | 39 | 239 | 0.1632 | 0.0000 | 0.0014 |
| 20MPa | ko00071 | fatty acid degradation | metabolism | lipid metabolism | 39 | 387 | 0.1008 | 0.1385 | 0.4441 |
| 40MPa | ko01230 | biosynthesis of amino acids | metabolism | global and overview maps | 115 | 876 | 0.1313 | 0.0006 | 0.0169 |
| 40MPa | ko01200 | carbon metabolism | metabolism | global and overview maps | 82 | 841 | 0.0975 | 0.5450 | 0.9994 |
| **Group** | **Pathway ID** | **Pathway Name** | **Level 1** | **Level 2** | **TCGN** | **TGN** | **Ratio** | **P value** | **Q value** |
| 40MPa | ko01240 | biosynthesis of cofactors | metabolism | global and overview maps | 76 | 963 | 0.0789 | 0.9860 | 0.9994 |
| 40MPa | ko00500 | starch and sucrose metabolism | metabolism | carbohydrate metabolism | 74 | 550 | 0.1345 | 0.0030 | 0.0380 |
| 40MPa | ko04146 | peroxisome | cellular processes | transport and catabolism | 59 | 491 | 0.1202 | 0.0585 | 0.2972 |
| 40MPa | ko00010 | glycolysis | metabolism | carbohydrate metabolism | 58 | 421 | 0.1378 | 0.0049 | 0.0564 |
| 40MPa | ko00520 | amino sugar and nucleotide sugar metabolism | metabolism | carbohydrate metabolism | 58 | 453 | 0.1280 | 0.0209 | 0.1558 |
| 40MPa | ko00230 | purine metabolism | metabolism | nucleotide metabolism | 56 | 515 | 0.1087 | 0.2248 | 0.6964 |
| 40MPa | ko00620 | pyruvate metabolism | metabolism | carbohydrate metabolism | 47 | 514 | 0.0914 | 0.7213 | 0.9994 |
| 40MPa | ko00250 | alanine, aspartate and glutamate metabolism | metabolism | amino acid metabolism | 46 | 289 | 0.1592 | 0.0007 | 0.0169 |
| 20MPa | GO:0016021 | integral component of membrane | cellular component | cellular anatomical entity | 317 | 8269 | 0.0383 | 0.1592 | 0.3923 |
| 20MPa | GO:0005524 | ATP binding | molecular function | binding | 175 | 3277 | 0.0534 | 0.0003 | 0.0059 |
| 20MPa | GO:0005634 | nucleus | cellular component | cellular anatomical entity | 128 | 4834 | 0.0265 | 1.0000 | 1.0000 |
| **Group** | **Pathway ID** | **Pathway Name** | **Level 1** | **Level 2** | **TCGN** | **TGN** | **Ratio** | **P value** | **Q value** |
| 20MPa | GO:0005737 | cytoplasm | cellular component | cellular anatomical entity | 110 | 2520 | 0.0437 | 0.0290 | 0.1808 |
| 20MPa | GO:0046872 | metal ion binding | molecular function | binding | 86 | 1728 | 0.0498 | 0.0516 | 0.2435 |
| 20MPa | GO:0016491 | oxidoreductase activity | molecular function | catalytic activity | 84 | 1556 | 0.0540 | 0.0095 | 0.0904 |
| 20MPa | GO:0008270 | zinc ion binding | molecular function | binding | 77 | 2765 | 0.0278 | 1.0000 | 1.0000 |
| 20MPa | GO:0003677 | DNA binding | molecular function | binding | 74 | 2892 | 0.0256 | 1.0000 | 1.0000 |
| 20MPa | GO:0005829 | cytosol | cellular component | cellular anatomical entity | 68 | 1058 | 0.0643 | 0.0000 | 0.0003 |
| 20MPa | GO:0005886 | plasma membrane | cellular component | cellular anatomical entity | 67 | 1277 | 0.0525 | 0.0020 | 0.0253 |
| 40MPa | GO:0016021 | integral component of membrane | cellular component | cellular anatomical entity | 351 | 8269 | 0.0424 | 0.7979 | 0.8958 |
| 40MPa | GO:0005524 | ATP binding | molecular function | binding | 212 | 3277 | 0.0647 | 0.0000 | 0.0004 |
| 40MPa | GO:0005634 | nucleus | cellular component | cellular anatomical entity | 163 | 4834 | 0.0337 | 1.0000 | 1.0000 |
| 40MPa | GO:0005737 | cytoplasm | cellular component | cellular anatomical entity | 142 | 2520 | 0.0563 | 0.0008 | 0.0165 |
| 40MPa | GO:0046872 | metal ion binding | molecular function | binding | 101 | 1728 | 0.0584 | 0.0290 | 0.1737 |
| 40MPa | GO:0008270 | zinc ion binding | molecular function | binding | 100 | 2765 | 0.0362 | 0.9997 | 1.0000 |
| 40MPa | GO:0003677 | DNA binding | molecular function | binding | 91 | 2892 | 0.0315 | 1.0000 | 1.0000 |
|  |  |  |  |  |  |  |  |  |  |
| **Group** | **Pathway ID** | **Pathway Name** | **Level 1** | **Level 2** | **TCGN** | **TGN** | **Ratio** | **P value** | **Q value** |
| 40MPa | GO:0016491 | oxidoreductase activity | molecular function | catalytic activity | 87 | 1556 | 0.0559 | 0.0925 | 0.3181 |
| 40MPa | GO:0005829 | cytosol | cellular component | cellular anatomical entity | 78 | 1058 | 0.0737 | 0.0000 | 0.0004 |
| 40MPa | GO:0022857 | transmembrane transporter activity | molecular function | transporter activity | 72 | 1786 | 0.0403 | 0.9630 | 1.0000 |

Abbreviations: **TCGN** – term candidate gene numbers, **TGN** – term gene numbers.
